# Bulk and single-cell transcriptome profiling reveal extracellular matrix mechanical regulation of lipid metabolism reprograming through YAP/TEAD4/ACADL axis in hepatocellular carcinoma

**DOI:** 10.7150/ijbs.82177

**Published:** 2023-04-09

**Authors:** Jingwei Cai, Tianyi Chen, Zhiyu Jiang, Jiafei Yan, Zhengtao Ye, Yeling Ruan, Liye Tao, Zefeng Shen, Xiao Liang, Yifan Wang, Junjie Xu, Xiujun Cai

**Affiliations:** 1 Key Laboratory of Laparoscopic Technology of Zhejiang Province, Department of General Surgery, Sir Run-Run Shaw Hospital, Zhejiang University School of Medicine, 310016, Hangzhou, China.; 2 Zhejiang Minimal Invasive Diagnosis and Treatment Technology Research Center of Severe Hepatobiliary Disease, Zhejiang Research and Development Engineering Laboratory of Minimally Invasive Technology and Equipment 310016, Hangzhou, China.; 3 Zhejiang University Cancer Center, 310058, Hangzhou, China.; 4 Liangzhu Laboratory, Zhejiang University Medical Center, 311121, Hangzhou, China.

**Keywords:** Hepatocellular carcinoma, extracellular matrix, ACADL, YAP

## Abstract

Emerging studies have revealed matrix stiffness promotes hepatocellular carcinoma (HCC) development. We studied metabolic dysregulation in HCC using the TCGA-LIHC database (n=374) and GEO datasets (GSE14520). HCC samples were classified into three heterogeneous metabolic pathway subtypes with different metabolic profiles: Cluster 1, an ECM-producing subtype with upregulated glycan metabolism; Cluster 2, a hybrid subtype with partial pathway dysregulation. Cluster 3, a lipogenic subtype with upregulated lipid metabolism; These three subtypes have different prognosis, clinical features and genomic alterations. We identified key enzymes that respond to matrix stiffness and regulate lipid metabolism through bioinformatic analysis. We found long-chain acyl-CoA dehydrogenase (ACADL) is a mechanoreactive enzyme that reprograms HCC cell lipid metabolism in response to extracellular matrix stiffness. ACADL is also regarded as tumor suppressor in HCC. We found that increased extracellular matrix stiffness led to activation of Yes-associated protein (YAP) and the YAP/TEA Domain transcription factor 4 (TEAD4) transcriptional complex was able to directly repress ACADL at the transcriptional level. The ACADL-dependent mechanoresponsive pathway is a potential therapeutic target for HCC treatment.

## Introduction

Hepatocellular carcinoma (HCC) is the third most common cause of cancer-related deaths worldwide^1^. This dismal outcome has been attributed to patients being diagnosed at late stages and the high frequency of metastasis in HCC. Even among patients with resectable HCC (most of them belonging to early stage), up to 70% will experience intra- or extra-hepatic recurrent metastasis after resection^2^. As a highly heterogeneous tumor, although some regulators attributing to HCC progression has been identified, the molecular mechanisms underlying the rapid cell proliferation and metastasis of HCC cells are largely unknown^3^. There is an urgent need for more complete understanding of the molecular mechanisms involved in deregulated HCC cell proliferation, which could help improve therapeutic strategies.

The majority of HCC develop in liver fibrosis or cirrhosis^4^, a condition in which altered biochemical and biophysical microenvironment initiates cancer onset and progression. Matrix stiffness is a characteristic of biophysical signals in the microenvironment and potently regulates cellular behaviors in various physio-pathological processes. Abnormal matrix stiffness plays a significant role during tumor progression. Increasing matrix stiffness is a strong predictor of HCC incidence, regulates HCC cell proliferation, chemotherapeutic response, invasion and metastasis^5^, and correlates with poor survival in HCC patients^6^,^7^. The signal from physical matrix stiffness is converted into various biochemical responses to drive malignant behaviors; among these, metabolic input that occurs during matrix stiffness-induced tumor progression is poorly understood.

Herein, we examined the specific contribution of lipid metabolic reprogramming to HCC progression triggered by matrix stiffness. Long-chain acyl-CoA dehydrogenase (ACADL) is a mitochondrial enzyme that catalyzes the initial step of fatty acid oxidation. Previous study has found that ACADL played a tumor-suppressor role in HCC^8^,^9^. Furthermore, ACADL is an essential mechano-mediator that reprograms HCC cell lipid metabolism. Collagen deposit indicates increased matrix stiffness in the tumor microenvironment. We found that in human HCC tissues, collagen content, a marker of increased matrix stiffness, and decreased expression of ACADL both predicted poor survival in HCC patients. These findings reveal that an ACADL-dependent mechanoresponsive pathway responds to increasing matrix stiffness (the biomechanical signals in the tumor microenvironment) and promotes HCC progression through lipid metabolic reprogramming, thereby providing a new therapeutic target for HCC treatment.

## Results

### Bulk Transcriptomic Profiling Revealed Evident Metabolic Dysregulation in HCCs

**Figure 1:**
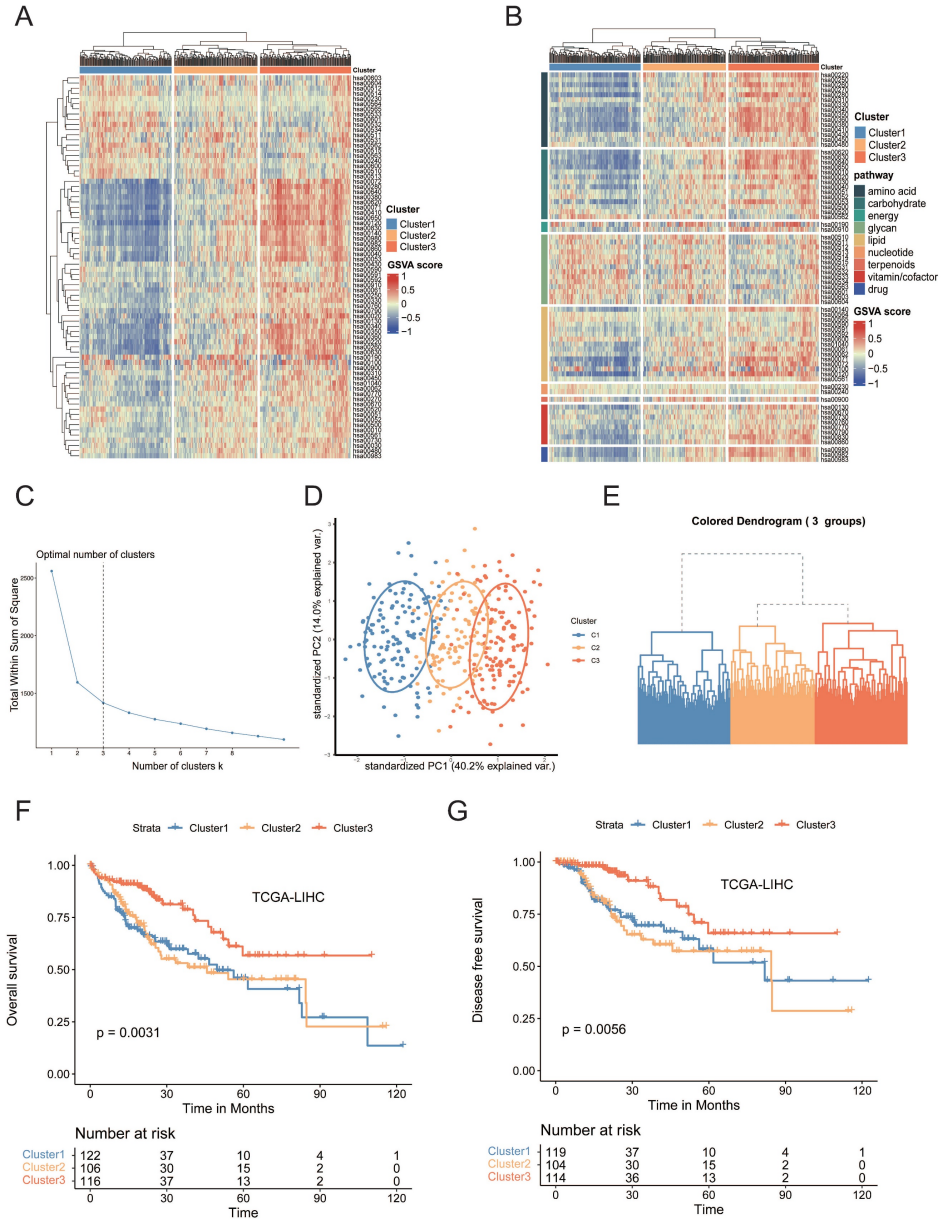
(A) Heatmap of metabolic-pathway-based GSVA analysis in TCGA-LIHC samples. (B) Heatmap of metabolic-pathway-based GSVA analysis according to the metabolic classes in TCGA-LIHC samples. (C) Determination of the optimal number of clusters (k). The elbow method shows *k* =3 as the optimal number of clusters. (D) The PCA plot of the samples from three different subtypes. The blue spots indicated the samples from cluster 1. The yellow spots indicated the samples from cluster 2, and the red spots indicated the samples from cluster 3. (E) Hierarchical clustering highlighting three different clusters. Euclidian distances and Ward's linkage were used. (F) Kaplan-Meier curves of OS among clusters in the TCGA-LIHC cohort. (G) Kaplan-Meier curves of DFS among clusters in the TCGA-LIHC cohort.

To investigate the mechanism behind metabolic reprogramming of HCCs, we selected 2886 human genes associated with 86 metabolic pathways from the Kyoto Encyclopedia of Genes and Genomes (KEGG) database (Table S1)^10^. To reveal the metabolic heterogeneity of HCCs, we estimated, based on gene set variation analysis (GSVA), the enrichment scores of 74 metabolic pathways in each sample from the Cancer Genome Atlas (TCGA) cohort, among which 12 pathways were filtered out due to relatively low sample size^11^. Based on the hierarchical clustering result, a three-cluster solution was found to be optimal for the TCGA dataset and was fitted in the clustering models. All 374 HCC tumors were classified into three heterogeneous subtypes based on the enrichment scores of 74 metabolic pathways, cluster 1 to cluster 3 (Figure 1A-E, Table S2). Cluster 1 (34.5% of all tumors, n=129), was characterized by the relative upregulation of glycan metabolism pathways, including N-Glycan biosynthesis, O-Glycan biosynthesis, and Chondroitin sulfate biosynthesis, which were all associated with the production of extracellular matrix (ECM), but the three major nutrients (carbohydrates, lipids and amino acids) metabolism pathway were downregulated. Cluster 3 (34.2% of all tumors, n=128), was characterized by remarkable upregulation of lipid, amino acid and carbohydrate metabolism pathways, including fatty acid degradation, arginine biosynthesis, histidine metabolism, and pyruvate metabolism. Cluster 2 (31.3% of all tumors, n=117) was characterized by the combined dysregulation of each major category. Similar patterns were observed in external independent cohorts using data from GSE14520 and ICGC) database (Figure S1A-S1D)^12^. Tumor purity is considered as a frequent contributing factor in the study of heterogeneity since variations in tumor purity can lead to skewed estimations of heterogeneity. Tumor purity analysis was performed by using “Estimate” R package. There was no statistically significant difference in tumor purity across the three clusters (Figure S1E). Principal component analysis (PCA) revealed the presence of three distinct transcriptomic clusters (Figure 1D). Furthermore, the three subtypes demonstrated distinct clinical characteristics (Figure S1F, Table S3). Significantly, the majority of patients of cluster 3 were male (p < 0.001) with a higher percentage of older patients (p < 0.05). Patients in cluster 1 had a significantly higher tumor grade percentage (p <0.001) and more advanced clinical stage (p < 0.05). Moreover, cluster 1 had significantly worse overall survival (OS) (p < 0.01) and disease-free survival (DFS) (p < 0.01) than cluster 3 (Figure 1F, G). Moreover, we next performed GSVA to figure out dynamics of biological processes and pathways for Hallmark gene sets of each cluster. GSVA/hallmark pathway analysis revealed significant upregulation of wnt β-catenin signaling, TGF-β signaling and PI3K/Akt mTOR signaling in cluster 1, implicating a variety of cancer-associated pathways were activated (Figure S1F). Collectively, our results demonstrated that the metabolic heterogeneity of HCCs fell into three metabolic phenotypes. ECM receptor relevant pathways were found significantly enriched and fatty acid metabolism pathways were downregulated in cluster 1 by different analysis approaches. Collectively, downregulation of fatty acid metabolism was correlated with the aggressiveness of tumors.

### Metabolic-Pathway-Based Clusters Show Distinct Genomic and Transcriptomic Characteristics

**Figure 2:**
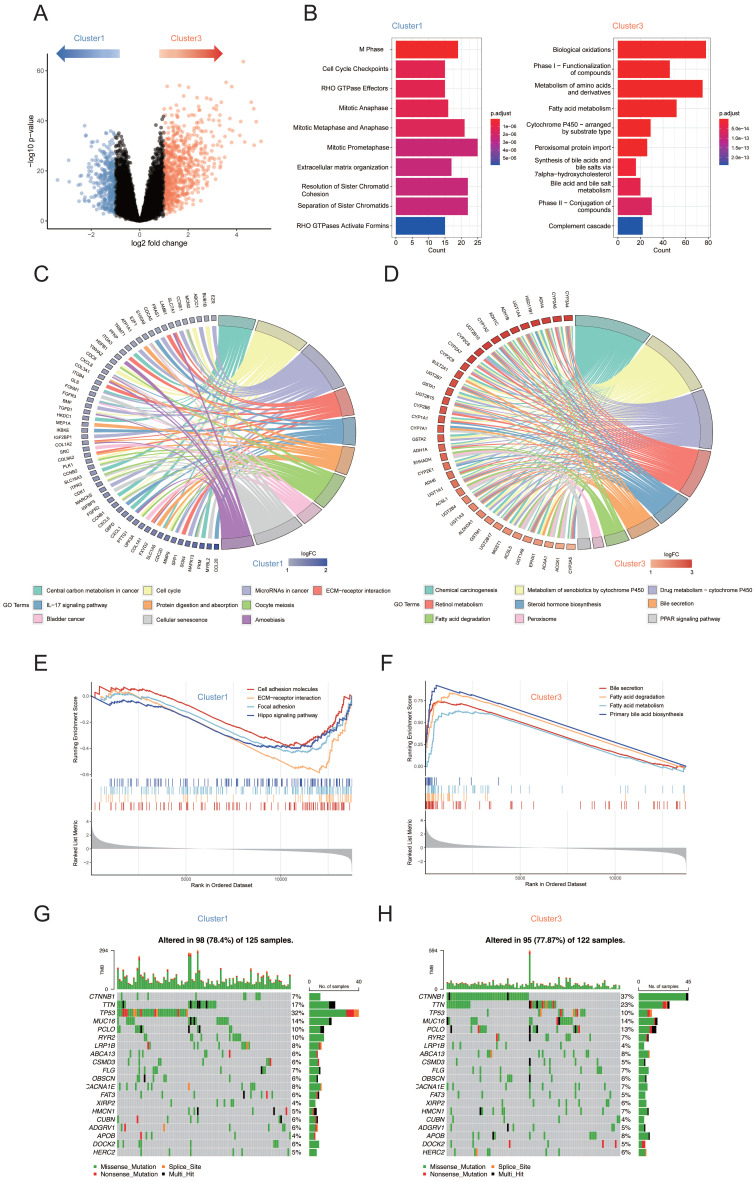
(A) Volcano plots showing genes that are differentially expressed in cluster 1 or cluster 3 samples. Blue dots indicate genes upregulated in cluster 1; red dots indicate genes upregulated in cluster 3. (B) Reactome enrichment analyses of genes upregulated in cluster 1 and cluster 3. (C) Circos plot showing top 10 KEGG pathway enriched in cluster 1. (D) Circos plot showing top 10 KEGG pathway enriched in cluster 3. (E) GSEA identified cell adhesion molecules, ECM-receptor interaction, focal adhesion and Hippo signaling pathway upregulated in cluster 1 subtype. (F) GSEA identified bile secretion, fatty acid degradation, fatty acid metabolism and primary bile acid biosynthesis pathway upregulated in cluster 3 subtype. (G) The waterfall plot showing the mutation distribution of the top 20 most frequently mutated genes in cluster 1. (H) The waterfall plot showing the mutation distribution of the top 20 most frequently mutated genes in cluster 3.

To determine whether cluster 1 tumors possess unique transcriptome programs that might suppress their metabolism phenotype, we constructed volcano plots to compare the gene expression in the samples from cluster 1 and cluster 3 (Figure 2A). To explore whether the differentially expressed genes between cluster 1 and cluster 3 that are related to specific functional features, we performed KEGG and REACTOME analysis for each differentially expressed gene group. Pathways of mitotic cell cycle checkpoints, cell cycle phase and extracellular matrix organization were found to be relatively enriched in cluster 1, while, as expected, gene sets related to the substance metabolic process such as metabolism of amino acids and derivatives, fatty acid metabolism and cytochrome P450 were found to be enriched in cluster 3 (Figure 2B). Similar results were obtained using KEGG analysis in circos plot. Categories related to tumorigenesis and development such as “Cell Cycle” and “ECM-receptor interaction” were enriched in cluster 1 (Figure 2C). Lipid-related metabolic pathways including retinol metabolism, steroid hormone biosynthesis, bile secretion and fatty acid degradation were enriched in cluster 3 (Figure 2D). Gene set enrichment analysis (GSEA)^13^ further demonstrated that fatty acid and bile acid related metabolic pathways were significantly upregulated in cluster 3 compared with cluster 1 whereas pathways included “focal adhesion”, “cell adhesion molecules”, “ECM-receptor interaction” and “Hippo signaling pathway” were significantly upregulated in cluster 1 compared with cluster 3 (Figure 2E, F). Furthermore, to investigate the difference of somatic variations between two subtypes, we used Maftools to access HCC driver genes and further analyzed the top 20 genes with the highest mutation frequency (Figure 2G, H). The results showed that there were significant differences in the mutation frequency of CTNNB1, TP53 in the cluster 1 and cluster 3 groups (Chi-square test, both p < 0.001; Table S4). Based on the results above, we found that the upregulation of ECM related pathways is typically accompanied by the downregulation of lipid metabolism pathway. Taken together, the biological process of lipid metabolism is potentially linked to ECM composition.

### Correlation between the pathways associated with cancer associated fibroblast (CAF) and fatty acid degradation

**Figure 3:**
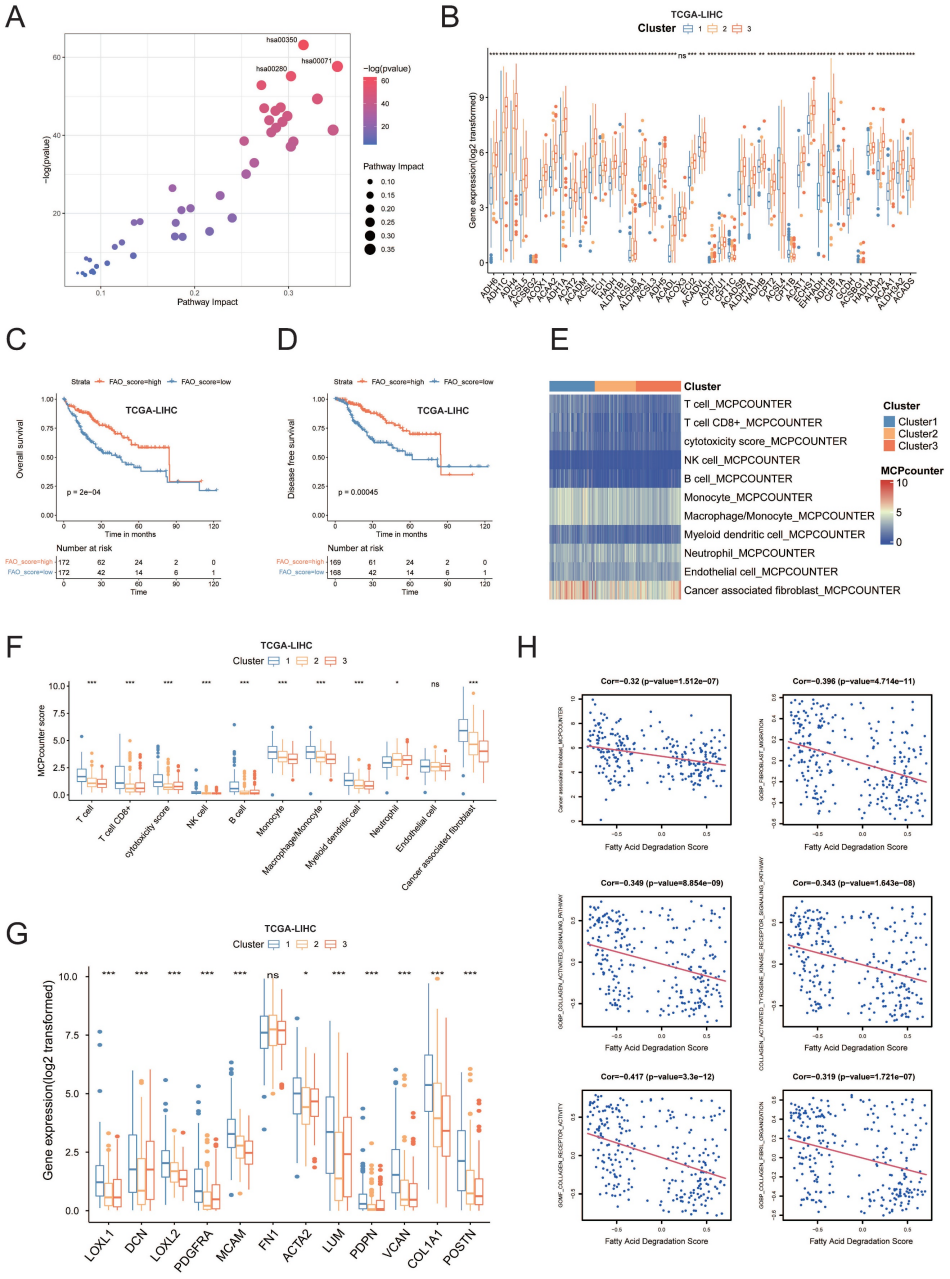
(A) Differential analysis of metabolic pathways suggested that 43 metabolic pathways were upregulated in cluster 3. (B) The boxplot showing the intensities of all of genes expression in hsa00071 geneset. Each boxplot shows the median and interquartile range (IQR, 25th-75th percentiles). The significance was determined using Wilcoxon rank-sum tests. (C) The OS rates were compared between the FAO-low enrichment score and FAO-high enrichment score groups. (D) The DFS rates were compared between the FAO-low enrichment score and FAO- high enrichment score groups. (E) Average microenvironment cell populations (MCP) among clusters to estimate the relative abundance of different cell populations in the TME displayed in a heatmap. (F) Quantification of MCP scores among clusters. (G) The boxplot outlining the expression of CAF markers COL1A1, POSTN, LUM, ACTA2, MCAM, PDGFRA, and genes for collagen synthesis enzymes among all clusters. (H) The association of the levels of fatty acid degradation enrichment score and fibroblast related pathways enrichment score was analyzed using Pearsons correlation analysis.

Pathway analysis demonstrated that these metabolic pathways were enriched with amino acid and lipid metabolism, particularly fatty acid degradation, which was also termed “fatty acid oxidation (FAO)” (hsa00071) (Figure 3A). Our metabolism-based classification reflected the expression levels of all genes from fatty acid degradation pathway, which were enriched in cluster 3 and most of genes showed significantly higher mRNA levels in cluster 3 compared with other subgroups (Figure 3B). Survival analyses showed that the patients with higher FAO pathway enrichment scores had better DFS and OS in comparison to those with low enrichment scores (p < 0.01; Figure 3C, D and Figure S1G). indicating that FAO pathway activation may inhibit HCC occurrence and development. The downregulation of FAO pathway could result in lipid accumulation, which generally leads to immunosuppressive effects and promotes tumor growth and metastasis^8^. We then investigated transcriptional patterns of different cell subsets in the microenvironment using the microenvironment cell populations-counter (MCP counter) (Figure 3E, F) and found significant differences between the cluster 1 and 3. Compared with patients in the cluster 3, the abundance of T-cell lineage, cytotoxic lymphocytes, NK cells, B cells, monocyte cells, macrophages, myeloid cells, and cancer associated fibroblasts (CAFs), were significantly higher in cluster 1, especially CAFs. Indeed, the patients from cluster 1 expressed high levels of CAF markers^14^ like COL1A1, POSTN, LUM, ACTA2, MCAM, PDGFRA, and genes for collagen synthesis enzymes (Figure 3G). In addition, the degree of CAF infiltration score was negatively correlated with the FAO enrichment score (Pearson's R = -0.32, p<0.001, Figure 3H). Meanwhile, FAO exhibited a significant negative correlation with fibroblast migration, collagen activated signaling pathway, collagen formation, collagen receptor activity and collagen biosynthetic process (Figure 3H).

Taken together, the biological process of fatty acid degradation was potentially linked to the functions of CAFs.

### Metabolic-Pathway-Based Subtypes of malignant and other cells in the tumor microenvironment based on scRNA-seq data

The single-cell resolution could help us to better understand the precise nature of subclonal diversity of tumors and the tumor microenvironment. Thus, we sought to determine whether the metabolic difference between the three clusters can be distinguished at the single-cell level. We first analyzed the GEO: GSE151530 database^15^ including 46 primary liver tumors samples and investigated the metabolic heterogeneity of cells in the tumor microenvironment.

**Figure 4:**
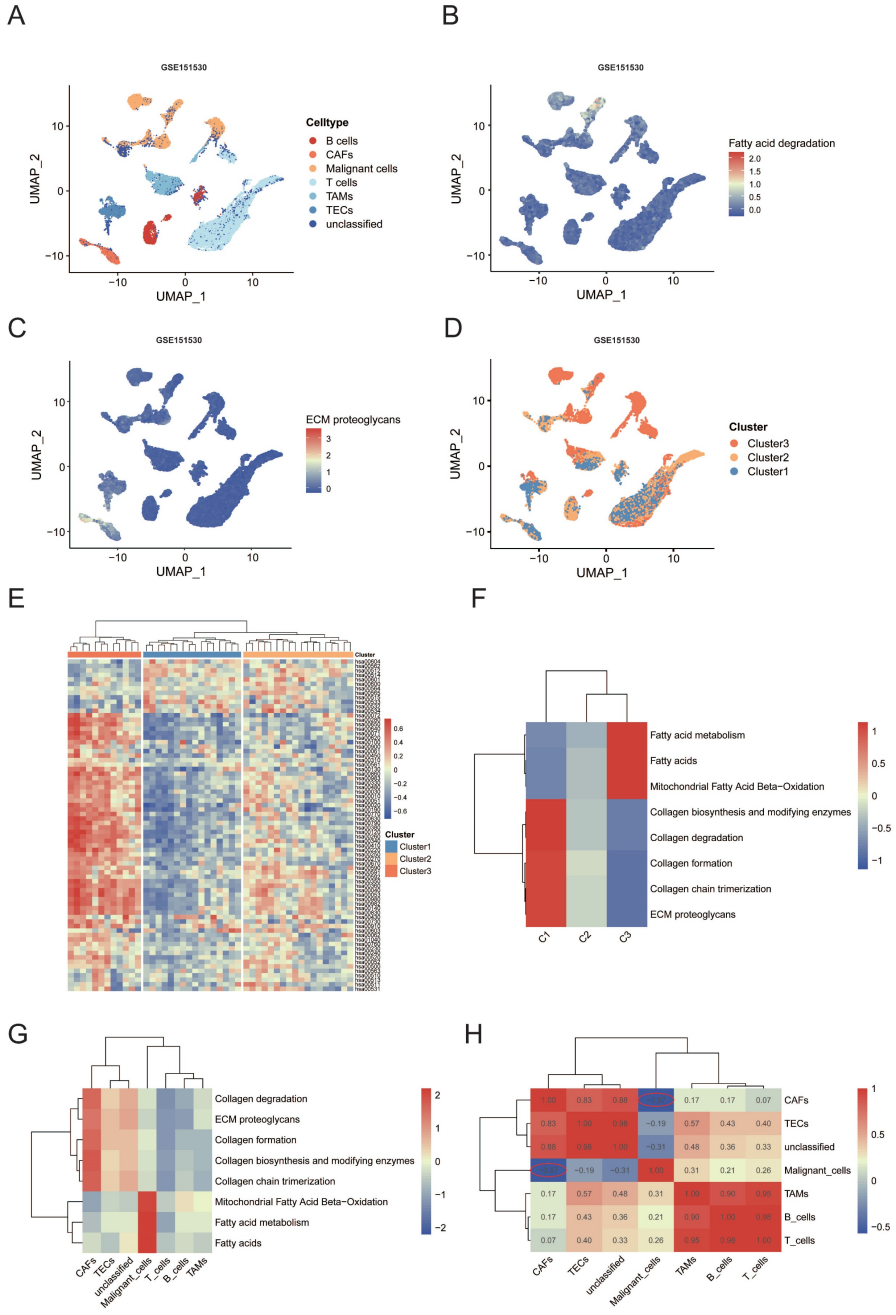
(A) Uniform manifold approximation and projection (UMAP) plot of seven cell types from liver tumors from GEO: GSE151530. (B) UMAP plot showing expression of fatty acid degradation pathway enriched in all cells. (C) UMAP plot showing expression of ECM proteoglycans pathway enriched in all cells. (D) UMAP plots showing the distribution of the three major clusters. (E) Heatmap representing the mean expression of metabolic-pathway-based GSVA analysis by three major clusters. (F) Functional enrichment analysis for the three clusters using “ReactomeGSA” package. (G) Functional enrichment analysis for the seven cell types using “ReactomeGSA” package. (H) The heatmap showing correlation between each pathway of seven cell types.

The cells were classified into seven major cell types according to the labels from the dataset, including T cells (n = 9286), malignant cells (n = 4784), unclassified cells (n = 3007), tumor-associated macrophages (TAMs) (n = 2184), B cells (n = 1718), tumor-associated endothelial cells (TECs)(n = 1580) and CAFs (n = 919) (Figure 4A). CellPhoneDB analysis based on receptor-ligand interactions was performed to reveal the cellular communication in HCC. Our analyses showed an apparently increased interactions of receptor-ligand pairs between Malignant cells and TAMs as well as CAFs, suggesting close cellcell communications among these two clusters (Figure S1H). We used the scMetabolism R package to visualize the metabolic diversity of single cells in uniform manifold approximation and projection (UMAP) representation. Results showed that fatty acid degradation pathway was mainly enriched in the malignant cells, while ECM proteoglycans pathway was enriched in CAFs (Figure 4B, C, Figure S1I). Furthermore, we performed GSVA to evaluate the pathway enrichment score of each patient at the bulk RNA-seq level. Interestingly, all of the patients were stably allocated into three metabolic subtypes based on the the same clustering approach (Figure 4D, E). Similar to the results of TCGA bulk RNA-sequencing, fatty acid degradation score was significantly upregulated in cluster 3 while O-glycan metabolism was upregulated in cluster 1 (Figure S1J, K, p < 0.001). Remarkably, the subclusters were highly patient specific, the proportion of each cell type varied greatly by cluster (Figure 4D, Figure S1L). To compare the fatty acid degradation score among malignant cells from different clusters, malignant cells were extracted for further analyses. Consistent results were shown that fatty acid degradation score was significantly upregulated in cluster 3 compared with cluster 1 (Figure S1M). ReactomeGSA analysis among all cell types and clusters revealed similar results of fatty acid metabolism and ECM relevant pathways. The collagen associated pathway like “Collagen formation” and “Collagen chain trimerization” was significantly enriched in CAFs and cluster 1. Lipid metabolism pathways were enriched significantly in malignant cells and cluster 3 (Figure 4F, G). The expression pattern of pathways above was clustered into a heat map according to the Pearson correlation. It could be observed that the level of pathways above showed a strong negative correlation (Pearson's R = -0.57, Figure 4H) between CAFs and malignant cells, which was consist with results from the bulk RNA-seq that fatty acid degradation was potentially associated with the functions of CAFs.

Together, both transcriptome analysis at single and bulk cell level identified the negative correlation between lipid metabolism and ECM remodeling.

### ACADL as a key enzyme of fatty metabolism is downregulated in HCCs

**Figure 5:**
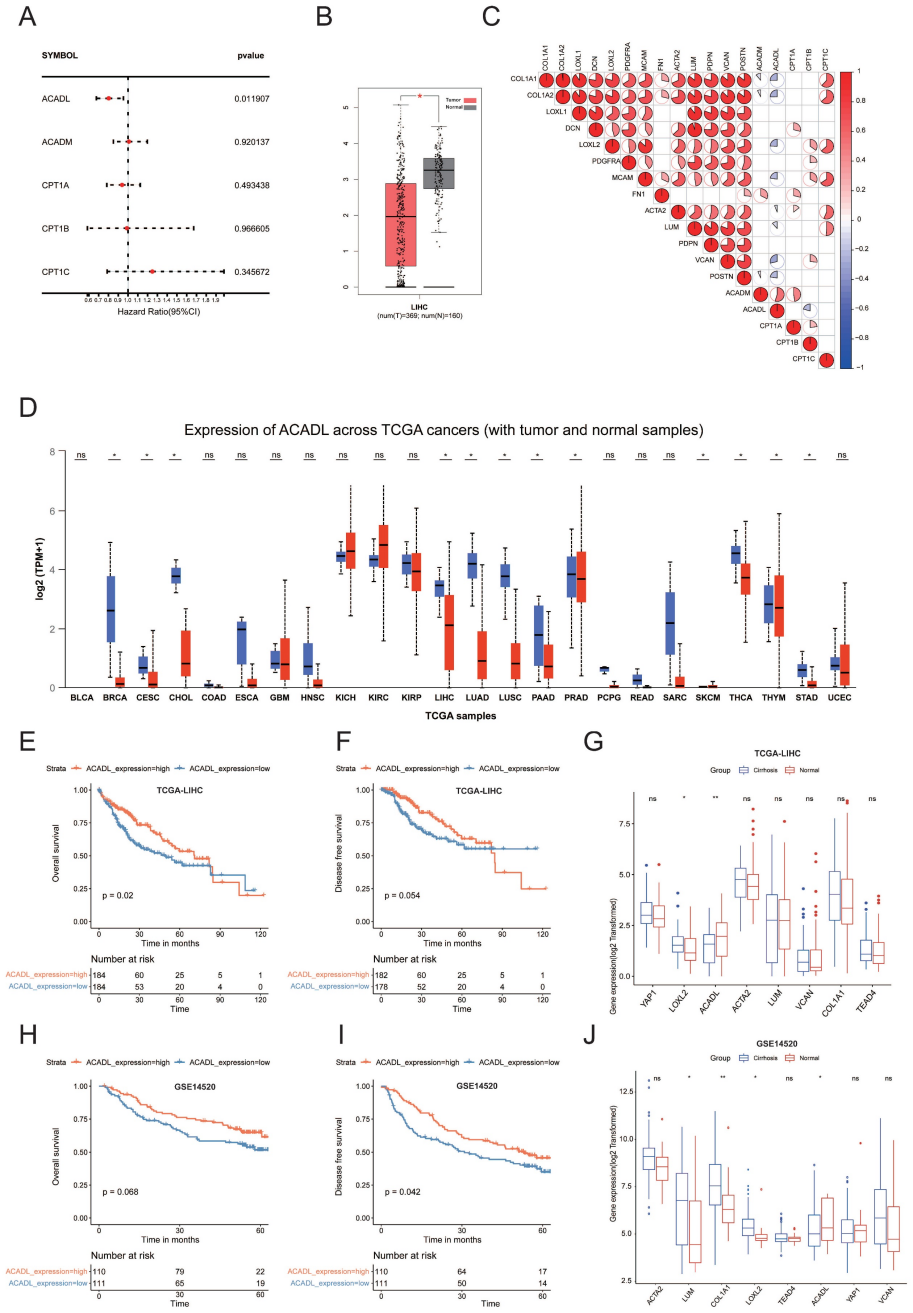
(A) Forest plot showing the hazard ratios from the univariate Cox regression analysis of five key enzyme of fatty acid degradation. (B) Box plots showed mRNA levels of ACADL in HCC tissues and noncancerous tissues in TCGA datasets from GEPIA. (C) The heat map shows that the mRNA level of ACADL was negatively related with the level of CAF-related genes. (D) Expression of ACADL across TCGA cancers (with tumor and normal samples) analyzed by UALCAN. (E) KM curve for OS of patients with high and low ACADL mRNA level by median value in TCGA datasets. (F) KM curve for DFS of patients with high and low ACADL mRNA level by median value in TCGA datasets. (G) Comparison of mRNA levels of ACADL and CAF marker genes between patients with and without liver cirrhosis in TCGA datasets. (H) KM curve for OS of patients with high and low ACADL mRNA level by median value in GSE14520 datasets. (I) KM curve for DFS of patients with high and low ACADL mRNA level by median value in GSE14520 datasets. (J) Comparison of mRNA levels of ACADL and CAF marker genes between patients with and without liver cirrhosis in GSE14520 datasets.

To explore the important molecules that may play roles in the fatty acid metabolism, we summarized the key enzymes of the top ten pathways highly expressed in cluster 3 (Table 1). Univariate Cox regression analysis of TCGA HCC dataset showed only FAO‐related gene (ACADL) remained significant (p <0.05) (Figure 5A, Table S5). ACADL, a key enzyme catabolizing the first step of FAO in mitochondria, was not only found decreased in HCC tissues (n = 369) compared with normal livers (n = 160) (Figure 5B). Interestingly, correlation heatmap showed that ACADL was negatively correlated with a variety of CAF and collagen related genes (Figure 5C). Moreover, ACADL was significantly decreased in various tumors (Figure 5D). Notably, HCC patients with low ACADL expression had poorer overall survival and disease-free survival than those with high ACADL expression (Figure 5E, F and Figure S2A). The tumor stage plot analysis using the UALCAN (http://ualcan.path.uab.edu/) from TCGA database further showed that ACADL expression was gradually decreased in higher HCC clinical stage (Figure S2B). According to the presence of cirrhosis, HCC patients were divided into 2 groups and the patients with stiffer hepatic background had lower ACADL expression (p < 0.01; Figure 5G). Importantly, similar results were observed in the external validation cohorts (Figure 5H-J). Herein, we hypothesize that stiffness may affect the lipid metabolism re-programing by regulating ACADL expression.

### Matrix stiffness modulates lipid metabolism re-programing via YAP-TEAD4-ACADL regulation

**Figure 6:**
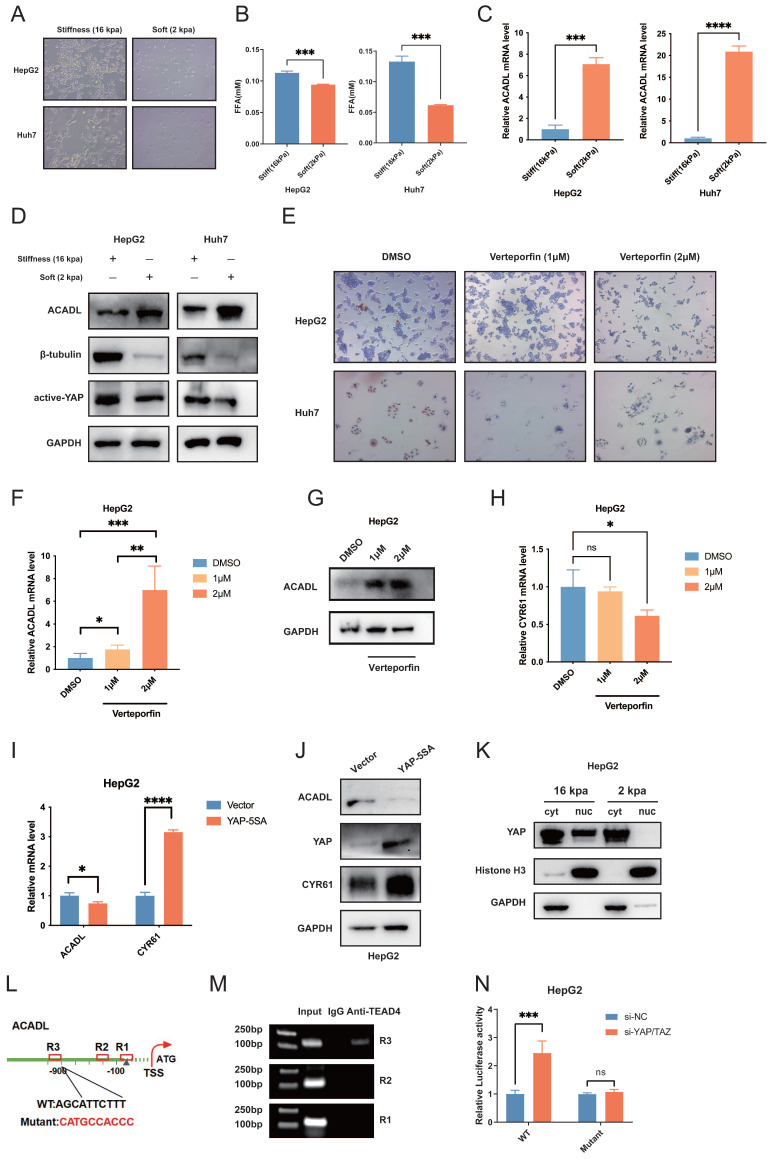
(A) HCC cells morphology was observed by light microscopy under different stiffnesses (2kpa &16kpa). (B) The level of free fatty acid release into cell culture medium under different stiffnesses (2kpa &16kpa) after 48 hours of culture. (C) qRT-PCR analysis of ACADL mRNA expression in HCC cells under different stiffnesses (2kpa &16kpa) after 48 hours of culture. (D) The expression level of ACADL, β-tubulin, and active yap were compared by western blotting, and GAPDH was used as a loading control. (E) Bright-field images of HCC cells after treated with DMSO or verteporfin of indicated concentration. (F) The mRNA expression level of ACADL of HepG2 cells after treated with DMSO or verteporfin of indicated concentration. (G) The protein level of ACADL of HepG2 cells after treated with DMSO or verteporfin of indicated concentration. (H) The mRNA level of CYR61 of HepG2 cells after treated with DMSO or verteporfin of indicated concentration. (I) The mRNA expression level of ACADL and CYR61 in YAP5SA overexpression HepG2 cells. (J) The western showing the expression levels of ACADL and CYR61 in YAP5SA overexpression HepG2 cells. (K) Cytosolic and nuclear proteins from HepG2 cells under different stiffnesses (2kpa &16kpa) were separated to detect expression of YAP by western blotting. Histone H3 and GAPDH were used as a loading control. (L) Schematic illustration of ACADL promoter region with potential TEAD4 binding sites (TBS). The WT and TBS mutant sequences were indicated. R1, R2, and R3 indicated Region1, Region2, and Region3 in ACADL promoter containing the potential TBS respectively. (M) A Chromatin immunoprecipitation (CHIP) analysis. HepG2 were used to extract cross-linked DNA, and CHIP was performed using anti-P300 and anti-RNAPol II. PCR was carried out using a primer designed according to the ACADL promoter. IgG CHIP was used as a negative control. (N) Luciferase analysis showing the effects of siRNA-YAP/TAZ on ACADL promoter R3 containing the WT or mutant TBS region in HepG2 cells.

The rigidity of the substrate that cells adhere can have a profound effect on cell morphology and gene expression. To test our hypothesis that stiffness may affect the lipid metabolism, HepG2, Huh7 and SK-Hep1 HCC cells were seeded on surface with micro-stiffnesses of 2 kPa and 16 kPa, respectively. HCC cells presented an invasive phenotype on stiff supports of 16 kPa, showing a protrusive appearance of polygonal cells with typical HCC cell morphology, while cells under 2 kPa stiff supports were spherical and appeared as small dots (Figure 6A, Figure S2C).

As shown in Figure 6B, liver cancer cells on high matrix stiffness at 16 kPa had increased free fatty acid levels in the media (p < 0.01, Figure 6B, Figure S2D) and markedly downregulated mRNA and protein levels of ACADL compared with the control group (p < 0.01; Figure 6C, D and Figure S2E, F). Previous studies have shown that signaling by diverse ECM components can trigger YAP/TAZ activity in different tissue types. For example, in the neonatal mouse heart, the ECM proteoglycan agrin binds to the dystrophin-glycoprotein complex (DGC) and induces YAP nuclear accumulation, promoting cardiomyocyte proliferation^16^,^17^. Moreover, the activation of the YAP transcription factor is a signature feature of CAFs. YAP function is required for CAFs to promote matrix stiffening, cancer cell invasion and angiogenesis^18^. In this study, upregulated protein levels of active YAP/TAZ were observed in high-stiffness matrices (Figure 6D).

To figure out the underlying mechanism of the downregulation of ACADL in HCC, we investigated the upstream regulator of ACADL by using hTFtarget and JASPAR2022 (Table S6, Figure S2G, H) and obtained 4 candidate TFs including FOXA2, HNF4A, TEAD4, YY1. Of note, YAP and TAZ are established TEAD4 co-transcriptional activators in the Hippo pathway. They are known to bind to TEAD family proteins, including TEAD4, to promote cell proliferation, growth and survival^19^. Because of the lack of DNAbinding motif in YAP, the transcription factors TEAD14 function as the major partners of YAP to regulate target gene expression. We hypothesized that YAP/TAZ could be activated by increased ECM stiffness, localizing to the nucleus and YAPTEAD4 complex directly targets ACADL, leading to reduced ACADL expression. To confirm this hypothesis, the expressions of ACADL, YAP1 and TEAD4 were re-analyzed in the TCGA database and it was found that ACADL was higher expressed while YAP1 and TEAD4 were lower expressed in cluster 3(Figure S2I). The expression of ACADL is negatively correlated with TEAD4 (Pearson's R = -0.477, p<0.001, Figure S2J). Verteporfin is a YAP inhibitor which disrupts YAP-TEAD interactions. Our results showed that HCC cells exhibited significantly lower levels of lipid accumulation when treated with increasing concentrations of verteporfin, suggesting that disrupting YAP-TEAD interactions could help utilizing lipid in HCC (Figure 6E, Figure S2K, p < 0.01). Our results also indicated that with an increase of verteporfin concentration, the ACADL mRNA and protein levels in HCC cells got increased (Figure 6F, G, Figure S2L, p < 0.01). Moreover, the transcriptional levels of YAP downstream genes CYR61, which was one of the well-recognized downstream target genes of YAP, were gradually decreased under increasing concentration of verteporfin treatment (Figure 6H, p < 0.01). Furthermore, the level of ACADL expression was then determined in YAP-overexpressed cells. Intriguingly, mRNA and protein levels of ACADL were both down-regulated in YAP5SA overexpression HepG2 and Huh7 cells (Figure 6I, J, and Figure S3A, B, both p < 0.01). Our cytoplasmic/nuclear protein extraction assay showed that less nuclear accumulation of YAP in cells on soft supports (Figure 6K). These results suggested that ACADL expression is negatively regulated by YAP at the transcriptional level.

To further illustrate the detailed mechanism, we recognized three conserved TEAD4 binding sites (AGCATTCTTT) in the promoter region of the human ACADL gene (Figure 6L). The chromatin immunoprecipitation (ChIP)PCR results indicated that TEAD4 might bind to the distal conserved sites (R3) of ACADL promoter (Figure 6M, Table S6). To further verify whether ACADL is the target gene of the YAPTEAD transcriptional complex, we generated two luciferase reporters driven by the wild type (WT) and TEAD4binding-deficient ACADL promoters (Figure 6K). Importantly, siRNAmediated YAP/TAZ knockdown significantly enhanced the transcriptional activity of WT ACADL promoter reporter in HepG2 and Huh7 cells, but had no effect on the mutant ACADL promoter reporter (Figure 6N, Figure S3C).

Collectively, these results suggested that YAP was activated in tumor cells by stiff substrates and consequently induce the repression of ACADL transcription via YAPTEAD4 complex binding to ACADL gene promoter.

### Soft substrates increase the mRNA and protein level of ACADL *in vivo*

**Figure 7:**
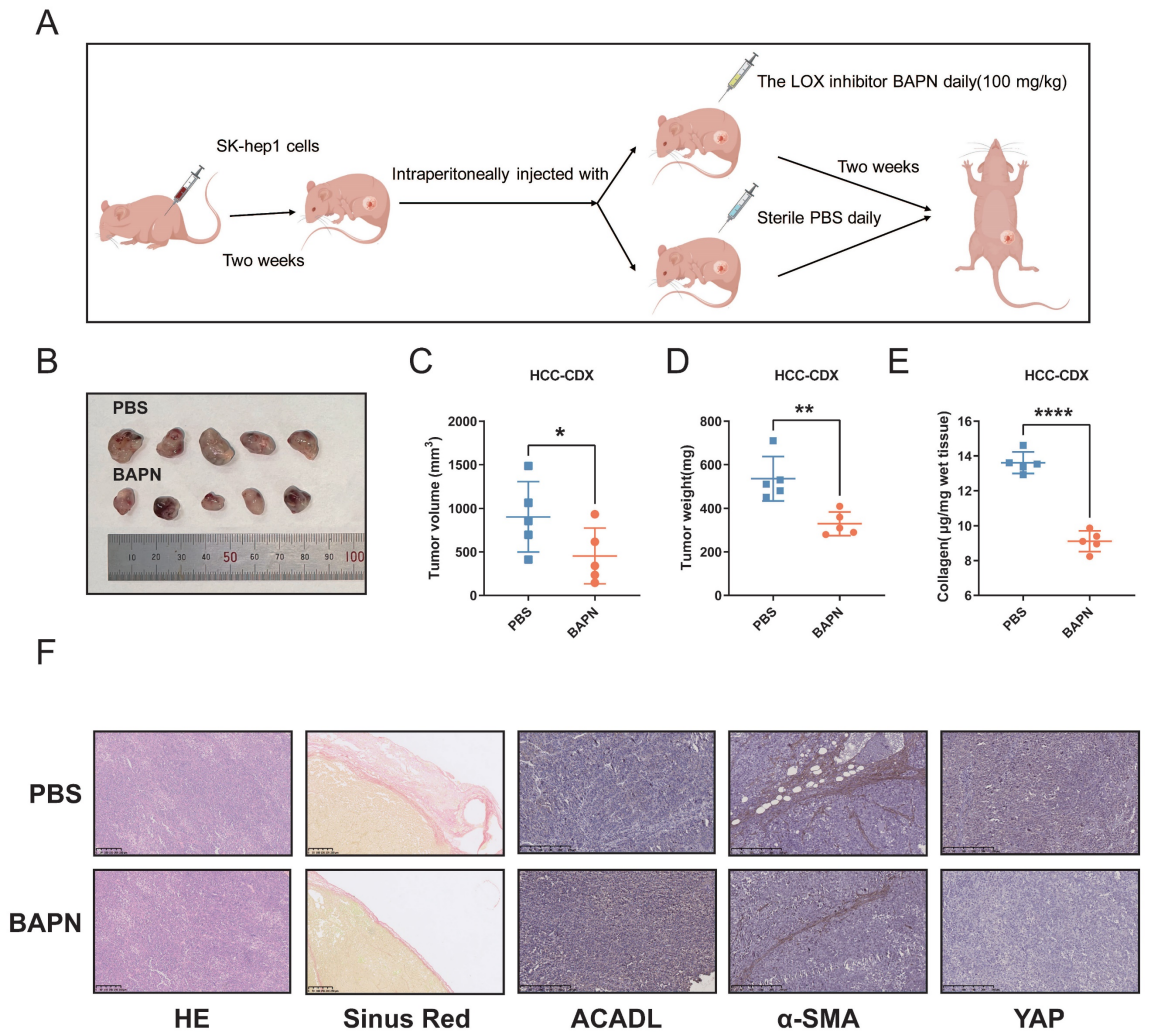
(A) Flow diagrams of animal experiments. (B) The SK-hep1 cells were injected subcutaneously into nude mice. A week later, the mice were administrated with β-aminopropionitrile (BAPN, 100 mg/kg) or PBS for indicated time. Representative image of the xenograft tumors obtained from the indicated groups. (C) Quantification for tumor volume of subcutaneous tumors in the indicated groups. (D) Quantification for tumor weight of subcutaneous tumors in the indicated groups. (E) Collagen content of xenograft tumors from indicated groups. (F) HE, Sirius red staining and expression of ACADL, a-SMA and YAP of subcutaneous tumors in the indicated groups were analyzed by immunohistochemistry.

The animal experimental workflow for our study is shown in a schematic representation (Figure 7A). β-aminopropionitrile (BAPN), a well-recognized inhibitor of LOX activity. Previous researches had proved that BAPN decreases tissue stiffness in mice with insulinomas^20^. The results indicated that the tumor volumes in BAPN group were significantly smaller than those in the control group (Figure 7B, C, p < 0.05). Additionally, the weight of tumors was significantly greater in the control group than the those in BAPN group (Figure 7D, p < 0.01). Hydroxyproline analysis revealed a significant decrease in collagen content in the BAPN group versus PBS control group (Figure 7E, p < 0.001). ACADL showed higher mRNA and protein levels in tumor tissues in BAPN group than PBS control group (Figure S3D, E, p < 0.01). In the tumors from the BAPN group, sirius red staining showed that collagen deposition was reduced in the same area of tumor stroma tissue. Moreover, ACADL expression was significantly increased, whereas that of YAP and α-SMA, which was considered as a marker gene of CAF, were markedly increased in BAPN group (Figure 7F).

### Increased collagen content and ACADL downregulation synergistically predict poor outcome in HCC patients

**Figure 8:**
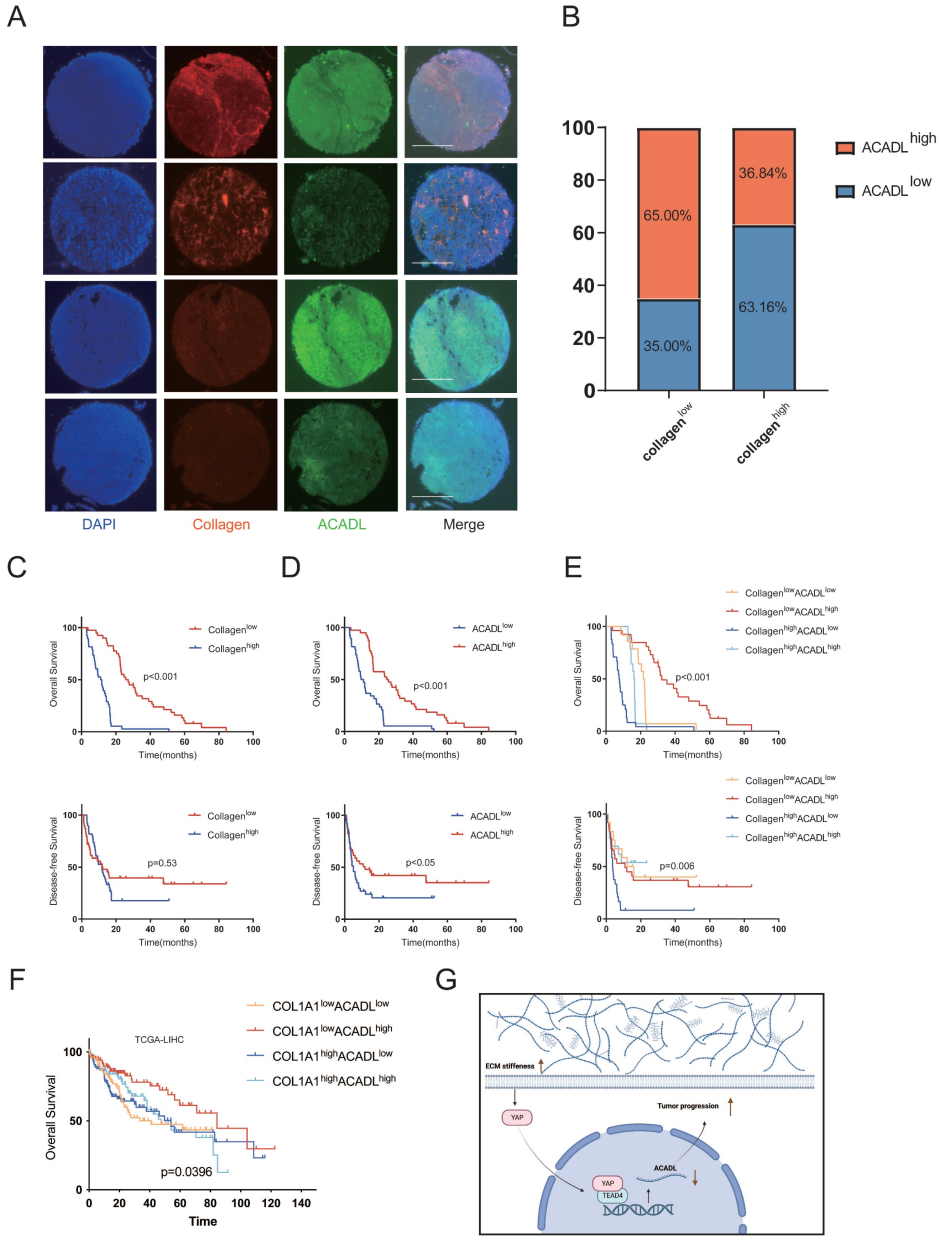
(A) Immunofluorescence staining of collagen type I and ACADL in HCC tissue chip. Scale bar represents 650 μm. (B) Patients divided into different proportions according to the level of collagen and ACADL. (C) Kaplan-Meier curve of OS and DFS for HCC patients grouped by intratumoral collagen content. (D) Kaplan-Meier curve of OS and DFS for HCC patients grouped by the protein levels of ACADL. (E) Kaplan-Meier curve of OS and DFS for HCC patients grouped by intratumoral collagen content and ACADL expression (collagen^low^/ACADL^low^ versus collagen^low^/ACADL^high^ versus collagen^high^/ACADL^low^ versus collagen^high^/ACADL^high^ tumors). (F) Kaplan-Meier curve of OS for TCGA-LIHC patients grouped by intratumoral collagen content and ACADL expression (COL1A1^low^/ACADL^low^ versus COL1A1^low^/ACADL^high^ versus COL1A1^high^/ACADL^low^ versus COL1A1^high^/ACADL^high^ tumors). Schematic of the proposed mechanism that an ACADL-dependent mechanotransduction pathway responsive to stiff matrix promotes HCC progression through lipid metabolic reprogramming. ECM, extracellular matrix

We explored the synergistic predictive value of collagen content and ACADL expression in tumor tissue on prognosis. In an independent cohort of 78 HCC patients, ACADL expression and intratumoral collagen content were measured by immunofluorescence in tissue microarray. The baseline characteristics of patients are summarized in Table S7. As an alternative marker of tissue stiffness, the mean fluorescence intensity of collagen type I was automatically quantified using Image J software to measure the collagen content. The collagen type I staining was found specifically in the stromal compartment of tumors while ACADL was preferentially distributed in the cytoplasm (Figure S3F). The collagen content was significantly negatively correlated with the level of ACADL expression in HCC (Figure 8A, B, p=0.013). The patients with stiffer tumors had a poor OS compared with patients with compliant tumors (p < 0.01; Figure 8C). Meanwhile, the patients with low ACADL expression had more unfavorable outcome than those with high ACADL expression (p < 0.05; Figure 8D). More significantly, the prognosis of HCC patients was stratified by the level of ACADL expression and collagen content. Patients with collagen^high^/ACADL^low^ tumors had markedly poor survival outcomes compared with patients with collagen^low^/ACADL^high^ tumors (p < 0.001, Figure 8E). Similarly, in the TCGA-LIHC cohort, tumors with collagen^high^/ACADL^low^ were associated with poor survival (p=0.0396, Figure 8F). These indicated the synergistic effect of increasing matrix stiffness and ACADL downregulation on HCC progression. Similar to the importance of HBV infection, the association between collagen^high^/ACADL^low^ tumors and poor prognosis was significant and independent of other clinicopathological parameters (Table S8).

In conclusion, this study provides a new mechanistic insight linking matrix stiffness-YAP-ACADL axis to lipid metabolic reprogramming and HCC progression, indicating that the ACADL-dependent mechanoresponsive pathway is a potential therapeutic target for HCC treatment (Figure 8G).

## Discussion

Matrix stiffness is a physical cue in the tumor environment that significantly increases carcinogenesis and tumor progression^21^. We attempted to explore the correlation between ECM and lipid metabolism through bioinformatics analysis, eventually targeting the key enzyme of lipid metabolism, ACADL, and analyzing the possible regulatory mechanisms. To the best of our knowledge, this is the first study that reveals that the ACADL-mediated mechanoresponsive pathway is downregulated by high matrix stiffness. This leads to lipid metabolic reprogramming, which in turn promotes HCC cell proliferation and metastasis. This study provided a new potential approach by enhancing ACADL expression/activity to reduce matrix stiffness-induced HCC progression.

HCC frequently develops and progresses in the fibrotic or cirrhotic liver with increased matrix rigidity, which has led to detrimental interactions between the altered biomechanical environment and HCC^22^. The increase in extra-/intra tumoral tissue rigidity or matrix stiffness plays a significant role in HCC progression^23^. Reportedly, the mechanical forces derived from matrix stiffness underlie the physio-pathological processes, including development^24^, inflammation^25^, and cancer^21^,^26^,^27^. The physical signal derived from matrix stiffness is a characteristic feature in the mechanical properties of extracellular matrix and transformed into intracellular biochemical responses to direct cancer cell behavior^28^,^29^. However, metabolic input during matrix stiffness induced tumor progression is largely unknown. Only a few studies have yet addressed the interconnection between matrix stiffening and tumor metabolic rewiring in cancer. This study explored the direct connection between matrix stiffness and lipid metabolic reprogramming. We used bioinformatic analysis and examined changes in lipid metabolism in HCC cells in response to matrix stiffness. Moreover, ACADL was identified as a key enzyme, initiating the FAO process, modulated by matrix stiffness. Lipid metabolic reprogramming influenced by matrix stiffness was interacted in an ACADL-dependent mechanoresponsive manner.

Changes in the actomyosin cytoskeleton also modulate the activity of the ubiquitously expressed paralogous factors YAP and TAZ, which have a central role in regulating transcription downstream of mechanical force generated by cell geometry, ECM stiffness, stretching and shear stress^30^. Previous study has indicated that Agrin was a tissue and ECM stiffness signal to activate YAP^31^. Agrin engages both integrins as well as Lrp4/MuSK for optimal activation of YAP in response to ECM stiffness^32^.

It is, however, very common for transcription factors to modulate the expression of their target genes both positively and negatively, depending on the context^33^. Repressive functions for YAP/TAZ were not well described. According to the gene expression microarray results performed in YAPoverexpressing cells, many genes were negatively regulated by YAP^34^. Besides the role of activating target gene transcription, the YAPTEAD complex has also been implicated to repress target gene transcription by recruiting members of the nucleosome remodelling and deacetylation (NuRD) complex to TSO (TEAD-SMAD-OCT4) elements^33^. Here, we identified ACADL as a target gene repressed by the YAPTEAD transcriptional complex. Future investigation will be interesting to identify more target genes repressed by the YAPTEAD4 complex, which may open an avenue for understanding the transcriptional repressor role of YAPTEAD.

Nevertheless, this study has some limitations. Firstly, C2 cluster was considered as an intermediate metabolic state, since C2 cluster has some characteristics of C1 cluster and C3 cluster. There was no detailed analysis in this article as this study focused on the inhibitory effects the extracellular matrix (ECM) on HCC lipid metabolism. Secondly, the direct molecular mechanism by which ACADL inhibit HCC carcinogenesis and progression was not determined. Although previous study has indicated that the tumor-suppressive effects of ACADL through inhibiting the Hippo/YAP signaling^35^. Moreover, regulation of extracellular matrix rigidity on lipid metabolism was not only exhibited at the transcriptional level, integration of multi-omics data may provide insights into comprehensive changes in biological pathways of lipid metabolism induced by ECM stiffness.

In this study, we demonstrated that subsets of HCCs were characterized by distinct transcriptomic signature depending on their metabolic features, indicating that different therapeutic strategies targeting metabolic vulnerabilities could have clinical benefits in subsets of HCC patients. Furthermore, this study also provides a new mechanistic linking matrix stiffness-ACADL axis to lipid metabolic reprogramming and HCC progression, indicating that the ACADL-dependent mechanoresponsive pathway is a potential therapeutic target for HCC treatment.

## Methods

### Ethical approval

This study was approved by the ethics committee of Sir Run Run Shaw Hospital. All animal studies were conducted according to the Association for the Assessment and Accreditation of Laboratory Animal Care and the Institutional Animal Care and Use Committee guidelines. All experiments were carried out in accordance with approved guidelines.

### Cell Culture and Transfections

The HCC cell line, Huh7, HepG2 and SK-Hep-1 were obtained from the American Type Culture Collection and were maintained in Dulbecco modified Eagle medium containing 10% (v/v) fetal bovine serum at 37°C in 5% CO2 condition. All cell lines were routinely tested to be negative for mycoplasma contamination. Transfection of siRNA and plasmids was performed using Lipofectamine 3000 Reagents (Thermo Fisher Scientific, Waltham, MA) following the manufacturer's instructions.

### Matrices with Different Stiffness for Cell Culture

As matrices with different surface micro-stiffnesses, we used 6-well CytoSoft® plates with two different micro-stiffnesses (2 kPa & 16 kPa) were coated with PureCol® Type I collagen, following manufacturer's instructions (Advanced Biomatrix, San Diego, CA, USA). Cells were seeded at a cell density of 2 × 105 cells/well for 48 h. Samples were collected for quantitative real time-polymerase chain reaction (qRT-PCR) or for western blotting analysis.

### RNA extraction and qRT-PCR

Total RNAs from HCC cells and samples were extracted using TRIzol reagent (Ambion, USA). cDNA was synthesized as well, using Hifair® II 1st Strand cDNA Synthesis SuperMix (Yeasen, Shanghai, China). Afterward, qPCR was conducted using the Hieff UNICON® qPCR SYBR Green Master Mix (Yeasen). Three independent replicates were conducted with every experiment. Then, the ∆∆Ct method was used for the relative calculation and quantification of mRNA. Glycer- aldehyde 3-phosphate dehydrogenase (GAPDH) was used as an internal control. Primer sequences used in this study are as depicted in Table S9 in Supporting Information.

### Western blotting analysis

The RIPA lysis buffer was used to extract total proteins. Sodium dodecyl sulfate-polyacrylamide gel electrophoresis (SDS-PAGE) was also used to separate extracted proteins. Subsequently, the PVDF membrane (Millipore) was used for protein transfer, following incubation of the transferred membrane with appropriate antibodies overnight at 4°C. The next day, enhanced chemiluminescence reagents (Fdbio Science) were used to detect the antigen-antibody complex on the membrane.

### Luciferase reporter assay

The promotor of ACADL containing the potential TEAD4 binding site was synthesized by TSINGKE Biological Technology (Beijing, China). The sequence was cloned into pGL3-basic (Promega, USA). The promotor of ACADL was inserted at the head of the firefly luciferase gene and the Renilla luciferase gene was used as an internal control. Cells transfected with ACADL promotor plasmid and Renilla luciferase plasmid were then transfected with si-YAP/TAZ and negative control after 48 hours. The Promega Dual-Luciferase Reporter assay system (Promega, USA) was used to measure the activities of firefly and Renilla luciferase.

### Human HCC Tissue Array and Immunofluorescence Assay

Tissue chip LVC1609, which consists of 80 paired HCC tumor and para-tumor samples, was purchased from Shanghai liaoding Biotech. The following antibodies were used to detect specific proteins: Collagen Type I (Mouse, 1:200, Proteintech, Cat No. CL594-67288), anti-ACADL (Rabbit, 1:300, Proteintech, Cat No. 17526-1-AP).

### Hydroxyproline analysis

Mouse tumor tissues were collected and hydroxyproline content was analyzed by the QuickZyme Total Collagen Assay from QuickZyme BioSciences. Briefly, the tumors were dried in an oven at 70°C for 48 hours and then hydrolyzed in 6 M HCL at 95°C for 20 hours. Then hydroxyproline content were measured following the manufacturer's protocol using collagen as standards.

### Measurement of lipids in medium

We treated HCC cells with verteporfin of different concentration gradient and cells were fixed under conventional six-well plate and subjected to oil red O staining after 48 hours of treatment. The non-esterified Free Fatty Acids (NEFA) was estimated in plasma by NEFA Colorimetric Assay Kit (E-BC-K013-M). NEFA ELISA kit (E-BC-K013-M) was purchased from Elabscience Biotechnology. Oil-Red-O staining was performed with Oil Red O Kit (G1262, Solarbio). Cells were washed with PBS for twice, and fixed with the fixative buffer for 30 min. Wash the cells with distilled water twice and then incubate in 60% isopropanol for 5 min. The newly prepared oil red O staining solution was added and soaked for 20 min. Mayer hematoxylin staining solution was added for 2 min. Discard the dye and wash it for 3 times. Oil-Red-O staining pictures were taken using a Zeiss inverted microscope. The Oil Red O Staining experiment was performed at least three independent repeat experiments. Quantifications were performed using Image J software.

### Survival analysis

Kaplan-Meier plots of OS and DFS were generated using the R package survival (http://cran.r-project.org/package=survival). A log-rank test p < 0.05 was used to define differences in survival time.

### Identification of differentially expressed genes

Differential pathway expression analyses between C1 and C3 and Differentially expressed genes (DEG) were performed by using limma package. Adjusted P-values (adjP) < 0.05 and log fold change (logFC) > 0.1 were considered to be significantly differentially expressed pathways. We considered logFC as “Pathway Impact” for differentially regulated pathways between two clusters.

### Metabolic Transcript Gene Set Variation Analysis (GSVA)

Pathway-level metabolic gene set enrichment analysis was performed using R Bioconductor package GSVA v1.32.0 function gsva() with parameters “method = gsva, min.sz = 5, max.sz = 500” using a log2(TPM + 1) transformed gene expression matrix^11^. GSVA pathway enrichment scores per sample were extracted and assessed for significance using R Bioconductor package limma v3.40.0. Pathway metabolite sets were constructed using the KEGG PATHWAY Database.

### Immune cell infiltration accessment

Microenvironment cell populations-counter (MCP-counter): MCP-counter is a computational Method based on the mean marker gene expression that is specifically expressed in the cell type^36^. The eight immune-cell lineage scores were estimated by using the R package MCP-counter algorithm.

### ScRNA-seq data processing

The GEO: GSE151530 dataset contains annotated cell types from each sample. We determined several cell types based on the annotation file in GEO: GSE151530 with the Seurat analysis package^37^. Specifically, T cells (CD4+ and CD8+), B cells, cancer-associated fibroblasts, tumor-associated macrophages, tumor-associated endothelial cells and epithelial cells could be recognized by uniform manifold approximation and projection (UMAP). The metabolism pathway enrichment of scRNA-seq data was analyzed through the ReactomeGSA R package^38^. The average gene expression level of each cell type was calculated using the “AverageExpression” function in Seurat.

CellphoneDB (v2.0.0) was used for cell-cell ligand receptor analysis.

### *In vivo* experiments

SK-Hep1 cells were inoculated subcutaneously in nude mice (n = 8) at 4-6 weeks of age. Two weeks after cells injection, the xenograft tumor models were successfully established in all nude mice. Xenograft tumor nude mice were treated with BAPN and PBS intraperitoneally, respectively. After another two weeks of treatment, the mice were sacrificed. Tumor volume was calculated using digital caliper measurements. Tumors were harvested and frozen in liquid nitrogen or fixed in 4% formalin immediately.

### Statistical analysis

Results are expressed as mean ± standard error of the mean. The Student t test was used for comparison between 2 groups. One-way analysis of variance (ANOVA) was used for comparisons among 3 or more groups. The Tukey test, Bonferroni test, or Dunnett test were used for post multiple comparisons between groups. The level of significance was P < .05 (*P < .05, **P < .01, and ***P < .001). The number of independent experiments was 3 (if not depicted otherwise). Calculations were performed using the GraphPad Prism Software (San Diego, CA).

## Supplementary Material

Supplementary figures and tables.

## Figures and Tables

**Table 1:** Top10 upregulated pathway in cluster 3 with key enzymes of each pathway.

pathway	pathway_name	key_enzyme
hsa00071	Fatty acid degradation - Homo sapiens (human)	ACADM, ACADL, CPT1A, CPT1B, CPT1C
hsa00120	Primary bile acid biosynthesis - Homo sapiens (human)	CYP7A1, CYP8B1, CYP27A1
hsa00280	Valine, leucine and isoleucine degradation - Homo sapiens (human)	BCAT1, BCAT2, BCKDHA,
hsa00350	Tyrosine metabolism - Homo sapiens (human)	TYR, TAT
hsa00053	Ascorbate and aldarate metabolism - Homo sapiens (human)	UGDH
hsa00380	Tryptophan metabolism - Homo sapiens (human)	TDO2, IDO1, IDO2, KMO
hsa00220	Arginine biosynthesis - Homo sapiens (human)	ASS1, ARG1, ARG2, ASL
hsa00650	Butanoate metabolism - Homo sapiens (human)	ACAT1, ACAT2
hsa00260	Glycine, serine and threonine metabolism - Homo sapiens (human)	SHMT1, SHMT2
hsa00410	beta-Alanine metabolism - Homo sapiens (human)	GAD1, GAD2, GADL1

## Abbreviations

HCC: hepatocellular carcinoma
KEGG: Kyoto Encyclopedia of Genes and Genomes
GSVA: gene set variation analysis
ECM: extracellular matrix
FAO: fatty acid oxidation
YAP: Yes-Associated Protein
BAPN: β-aminopropionitrile
