# Supplementary material for: Bulk and single-cell transcriptome profiling reveal extracellular matrix mechanical regulation of lipid metabolism reprograming through YAP/TEAD4/ACADL axis in hepatocellular carcinoma
Source: Int J Biol Sci. 2023 Apr 9;19(7):2114–31. doi: 10.7150/ijbs.82177 (PMC10158031; doi:10.7150/ijbs.82177)
Supplement: Supplementary file 1 — Supplementary figures and tables. [file ijbsv19p2114s1.pdf]

Figure S1

(A) Heatmap of metabolic-pathway-based GSVA analysis in GSE14520 cohort.
(B) Heatmap of metabolic-pathway-based GSVA analysis in ICGC cohort.
(C) Kaplan-Meier curves of OS among clusters in the GSE14520 cohort.
(D) Kaplan-Meier curves of DFS among clusters in the GSE14520 cohort.
(E) Violin plot for the tumor purity in three different subtypes.
(F) Heatmap depicting the normalized enrichment scores of 50 hallmark pathways among clusters.
(G) Kaplan-Meier curves of OS between the FAO-low enrichment score and FAO-high enrichment score groups in ICGC cohort.
(H) Heatmap describing all interaction numbers of different celltypes obtained with CellPhoneDB.
(I) Boxplot showing expression level of fatty acid degradation and ECM proteoglycans pathway enriched in all cells.
(J) Boxplot for the expression of ECM proteoglycans pathway score among clusters.
(K) Boxplot for the expression of fatty acid degradation pathway score among clusters.
(L) Cell type distributions in each sub-cluster.
(M) Violin plot for the expression of fatty acid degradation pathway score among malignant cells from clusters.

Figure S2

(A) KM curve for OS of patients with high and low ACADL mRNA level by median value in ICGC datasets.
(B) ACADL expression and its association with tumor grade in HCC were analyzed by UALCAN.
(C) SK-Hep1 cells morphology was observed by light microscopy under different stiffnesses (2kpa &16kpa).
(D) The level of free fatty acid release into cell culture medium under different stiffnesses (2kpa &16kpa) after 48 hours of culture in SK-Hep1 cells.
(E) qRT-PCR analysis of ACADL mRNA expression in HCC cells under different stiffnesses (2kpa &16kpa) after 48 hours of culture in SK-Hep1 cells.
(F) The expression level of ACADL, β-tubulin, and active yap in SK-Hep1 cells were compared by western blotting, and GAPDH was used as a loading control.
(G) Venn diagram showing the overlaps of two transcription factors (TF) list.
(H) Two TEAD4 motif sequence predicted by JASPAR.
(I) The boxplot outlining the expression of YAP1, ACADL and TEAD4 among all clusters.
(J) Pearson's correlation analysis between the mRNA level of ACADL and TEAD4 in TCGA-LIHC dataset.
(K) Small positive spots detected with oil red O staining in the cytoplasm were calculated after cells treated with DMSO or verteporfin of indicated concentration.

(L) The protein level of ACADL of Huh7 cells after treated with DMSO or verteporfin of indicated concentration.

Figure S3

(A) The mRNA expression level of ACADL and CYR61 in YAP-5SA overexpression Huh7 cells.
(B) The western showing the expression levels of ACADL and CYR61 in YAP-5SA overexpression Huh7 cells.
(C) Luciferase analysis showing the effects of siRNA-YAP/TAZ on ACADL promoter R3 containing the WT or mutant TBS region in Huh7 cells.
(D) ACADL mRNA level of cell-derived xenograft (CDX) models in the indicated groups were analysed by qPCR.
(E) ACADL protein expression of CDX models in the BAPN and PBS group tissues detected by western blot.
(F) Immunofluorescence staining of collagen type I and ACADL in HCC tissue chip. Scale bar represents 100 μm.

FigureS1

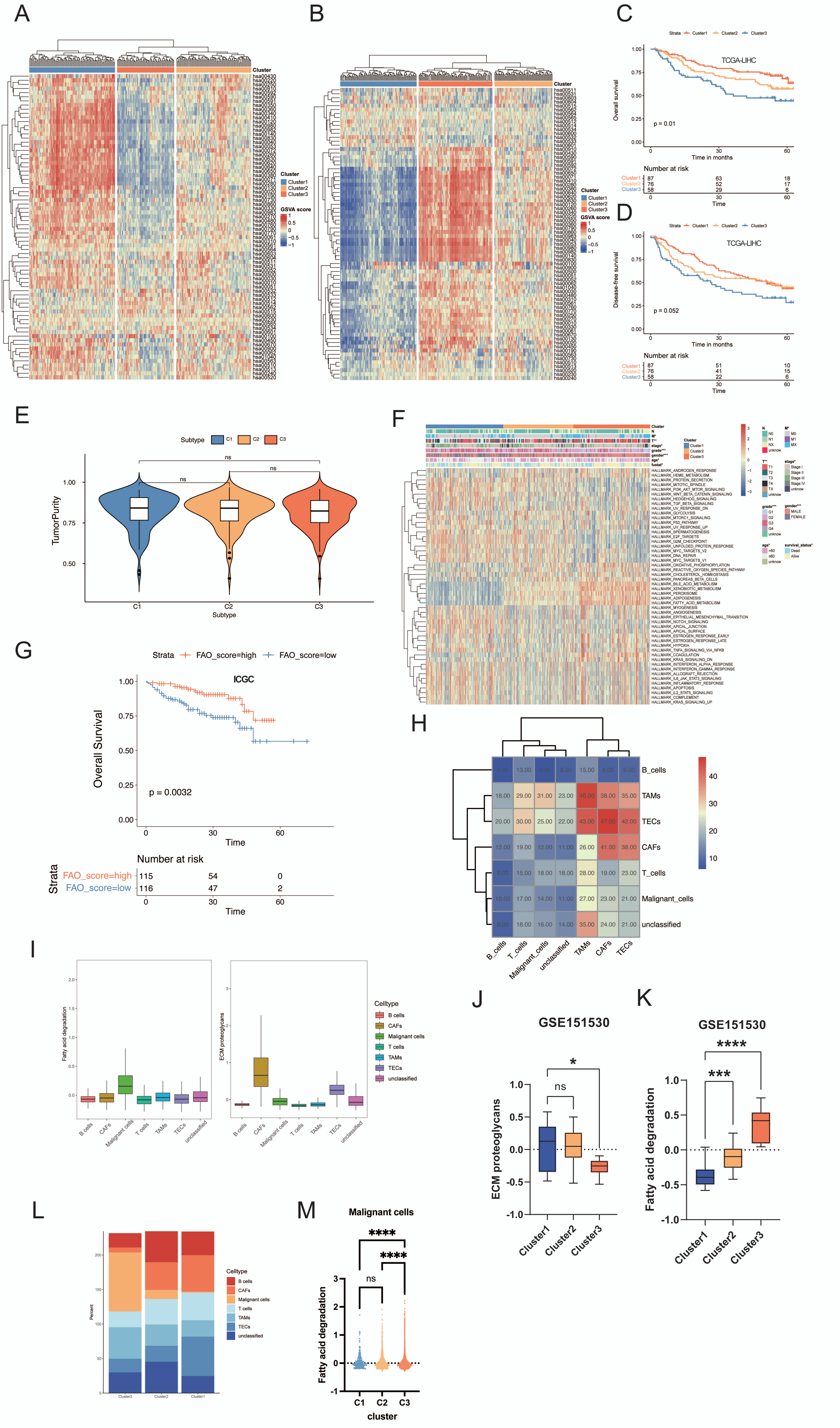

A
B
C
D
E
F
G
H
I
J
K
L
M
Cluster
Cluster1
Cluster2
Cluster3
GSVA score
TCGA-LIHC
Overall survival
Disease-free survival
Time in months
Number at risk
Time
p = 0.01
p = 0.052
Strata
Subtype
C1
C2
C3
TumorPurity
ns
FAO_score=high
FAO_score=low
ICGC
Overall Survival
p = 0.0032
B_cells
TAMs
TECs
CAFs
T_cells
Malignant_cells
unclassified
Fatty acid degradation
ECM proteoglycans
Celltype
B cells
Malignant cells
T cells
GSE151530
Percent
Malignant cells
cluster

FigureS2

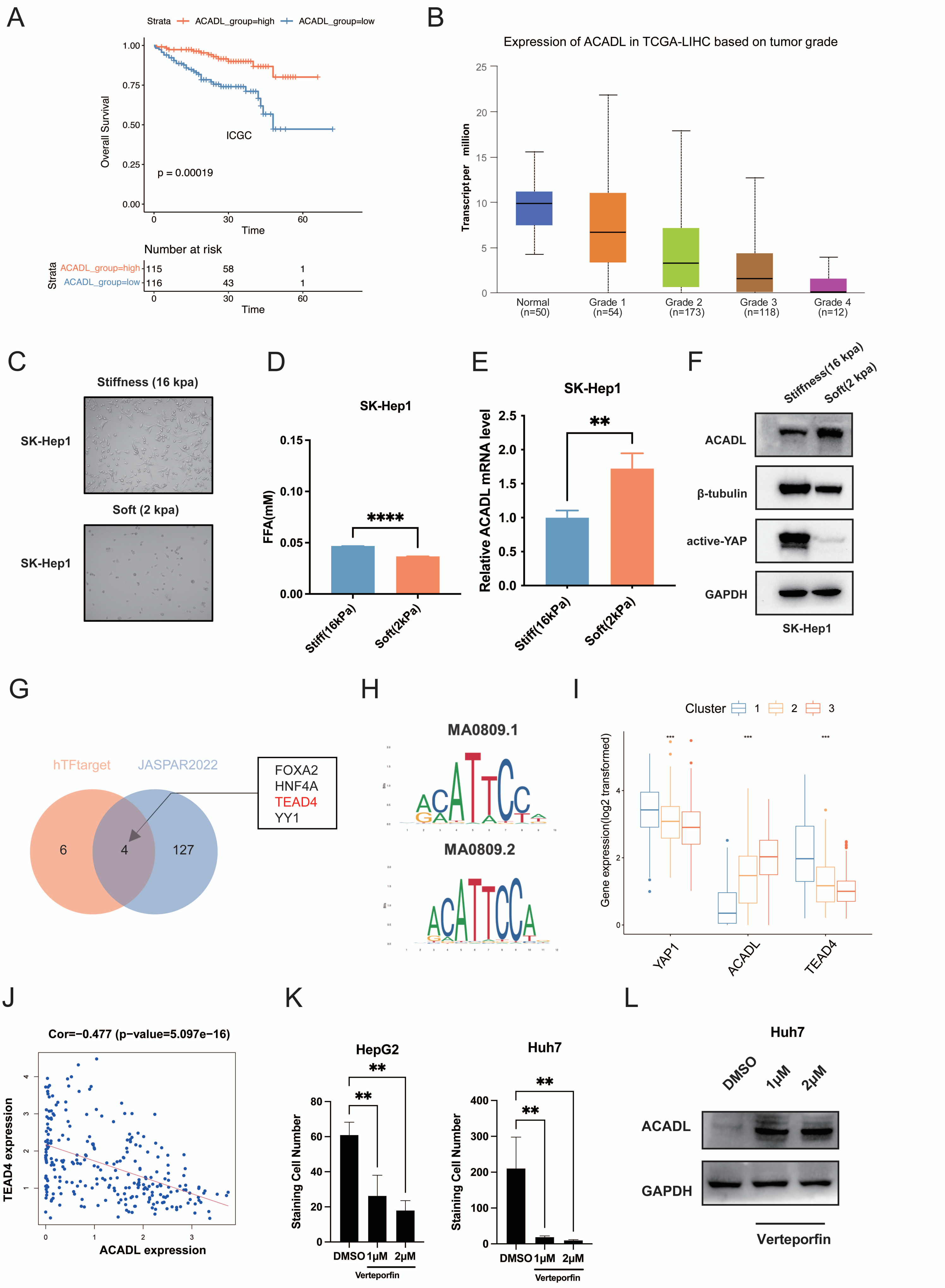
A
Strata ACADL_group=high ACADL_group=low
Overall Survival
ICGC
p = 0.00019
Time
Number at risk
ACADL_group=high 115 58 1
ACADL_group=low 116 43 1
B
Expression of ACADL in TCGA-LIHC based on tumor grade
Transcript per million
Normal (n=50)
Grade 1 (n=54)
Grade 2 (n=173)
Grade 3 (n=118)
Grade 4 (n=12)
C
Stiffness (16 kpa)
SK-Hep1
Soft (2 kpa)
SK-Hep1
D
SK-Hep1
FFA(mM)
****
Stiff(16kPa)
Soft(2kPa)
E
SK-Hep1
Relative ACADL mRNA level
**
Stiff(16kPa)
Soft(2kPa)
F
Stiffness(16 kpa)
Soft(2 kpa)
ACADL
β-tubulin
active-YAP
GAPDH
SK-Hep1
G
hTFtarget
JASPAR2022
6
4
127
FOXA2
HNF4A
TEAD4
YY1
H
MA0809.1
MA0809.2
I
Cluster 1 2 3
Gene expression(log2 transformed)
YAP1
ACADL
TEAD4
J
Cor=−0.477 (p−value=5.097e−16)
TEAD4 expression
ACADL expression
K
HepG2
Huh7
Staining Cell Number
**
**
DMSO 1μM 2μM
Verteporfin
L
Huh7
DMSO
1μM
2μM
ACADL
GAPDH
Verteporfin

FigureS3

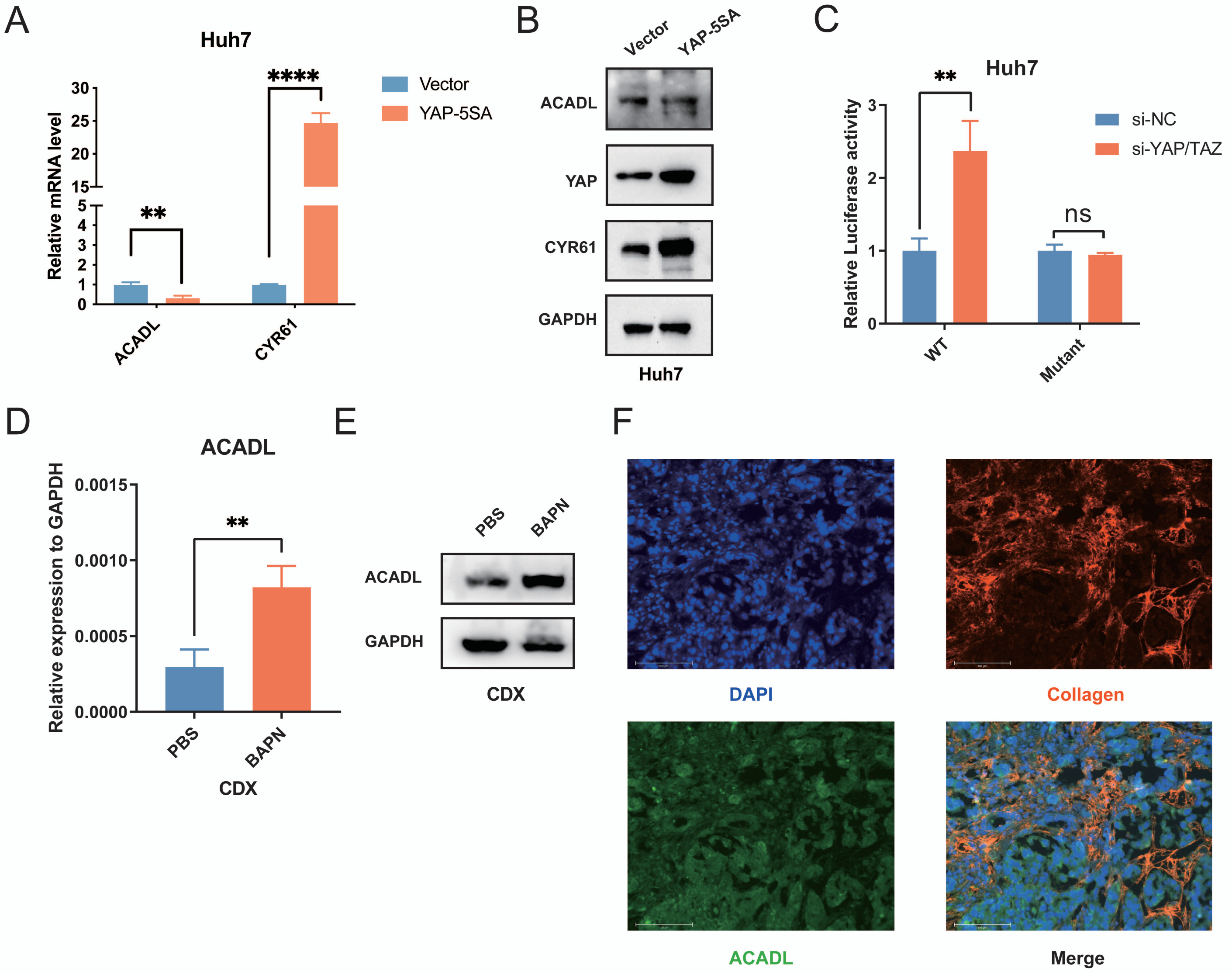
A
Huh7
Relative mRNA level
30
25
20
15
5
4
3
2
1
0
**
****
Vector
YAP-5SA
ACADL
CYR61
B
Vector
YAP-5SA
ACADL
YAP
CYR61
GAPDH
Huh7
C
Huh7
**
ns
si-NC
si-YAP/TAZ
Relative Luciferase activity
3
2
1
0
WT
Mutant
D
ACADL
Relative expression to GAPDH
0.0015
0.0010
0.0005
0.0000
**
PBS
BAPN
CDX
E
PBS
BAPN
ACADL
GAPDH
CDX
F
DAPI
Collagen
ACADL
Merge

Table S1: KEGG Human Metabolic Pathway Gene List, Related to Figure 1.

pathway_id	pathway_name
hsa00010	Glycolysis / Gluconeogenesis - Homo sapiens (human)
hsa00020	Citrate cycle (TCA cycle) - Homo sapiens (human)
hsa00030	Pentose phosphate pathway - Homo sapiens (human)
hsa00040	Pentose and glucuronate interconversions - Homo sapiens (human)
hsa00051	Fructose and mannose metabolism - Homo sapiens (human)
hsa00052	Galactose metabolism - Homo sapiens (human)
hsa00053	Ascorbate and aldarate metabolism - Homo sapiens (human)
hsa00061	Fatty acid biosynthesis - Homo sapiens (human)
hsa00062	Fatty acid elongation - Homo sapiens (human)
hsa00071	Fatty acid degradation - Homo sapiens (human)
hsa00072	Synthesis and degradation of ketone bodies - Homo sapiens (human)
hsa00100	Steroid biosynthesis - Homo sapiens (human)
hsa00120	Primary bile acid biosynthesis - Homo sapiens (human)
hsa00130	Ubiquinone and other terpenoid-quinone biosynthesis - Homo sapiens (human)
hsa00140	Steroid hormone biosynthesis - Homo sapiens (human)
hsa00190	Oxidative phosphorylation - Homo sapiens (human)
hsa00220	Arginine biosynthesis - Homo sapiens (human)
hsa00230	Purine metabolism - Homo sapiens (human)
hsa00232	Caffeine metabolism - Homo sapiens (human)
hsa00240	Pyrimidine metabolism - Homo sapiens (human)
hsa00250	Alanine, aspartate and glutamate metabolism - Homo sapiens (human)
hsa00260	Glycine, serine and threonine metabolism - Homo sapiens (human)
hsa00270	Cysteine and methionine metabolism - Homo sapiens (human)
hsa00280	Valine, leucine and isoleucine degradation - Homo sapiens (human)
hsa00290	Valine, leucine and isoleucine biosynthesis - Homo sapiens (human)
hsa00310	Lysine degradation - Homo sapiens (human)
hsa00330	Arginine and proline metabolism - Homo sapiens (human)
hsa00340	Histidine metabolism - Homo sapiens (human)
hsa00350	Tyrosine metabolism - Homo sapiens (human)
hsa00360	Phenylalanine metabolism - Homo sapiens (human)
hsa00380	Tryptophan metabolism - Homo sapiens (human)
hsa00400	Phenylalanine, tyrosine and tryptophan biosynthesis - Homo sapiens (human)
hsa00410	beta-Alanine metabolism - Homo sapiens (human)
hsa00430	Taurine and hypotaurine metabolism - Homo sapiens (human)
hsa00440	Phosphonate and phosphinate metabolism - Homo sapiens (human)
hsa00450	Selenocompound metabolism - Homo sapiens (human)
hsa00471	D-Glutamine and D-glutamate metabolism - Homo sapiens (human)
hsa00472	D-Arginine and D-ornithine metabolism - Homo sapiens (human)
hsa00480	Glutathione metabolism - Homo sapiens (human)
hsa00500	Starch and sucrose metabolism - Homo sapiens (human)
hsa00510	N-Glycan biosynthesis - Homo sapiens (human)

hsa00511	Other glycan degradation - Homo sapiens (human)
hsa00512	Mucin type O-glycan biosynthesis - Homo sapiens (human)
hsa00513	Various types of N-glycan biosynthesis - Homo sapiens (human)
hsa00514	Other types of O-glycan biosynthesis - Homo sapiens (human)
hsa00515	Mannose type O-glycan biosynthesis - Homo sapiens (human)
hsa00520	Amino sugar and nucleotide sugar metabolism - Homo sapiens (human)
hsa00524	Neomycin, kanamycin and gentamicin biosynthesis - Homo sapiens (human)
hsa00531	Glycosaminoglycan degradation - Homo sapiens (human)
hsa00532	Glycosaminoglycan biosynthesis - chondroitin sulfate / dermatan sulfate - Homo sapiens (human)
hsa00533	Glycosaminoglycan biosynthesis - keratan sulfate - Homo sapiens (human)
hsa00534	Glycosaminoglycan biosynthesis - heparan sulfate / heparin - Homo sapiens (human)
hsa00561	Glycerolipid metabolism - Homo sapiens (human)
hsa00562	Inositol phosphate metabolism - Homo sapiens (human)
hsa00563	Glycosylphosphatidylinositol (GPI)-anchor biosynthesis - Homo sapiens (human)
hsa00564	Glycerophospholipid metabolism - Homo sapiens (human)
hsa00565	Ether lipid metabolism - Homo sapiens (human)
hsa00590	Arachidonic acid metabolism - Homo sapiens (human)
hsa00591	Linoleic acid metabolism - Homo sapiens (human)
hsa00592	alpha-Linolenic acid metabolism - Homo sapiens (human)
hsa00600	Sphingolipid metabolism - Homo sapiens (human)
hsa00601	Glycosphingolipid biosynthesis - lacto and neolacto series - Homo sapiens (human)
hsa00603	Glycosphingolipid biosynthesis - globo and isoglobo series - Homo sapiens (human)
hsa00604	Glycosphingolipid biosynthesis - ganglio series - Homo sapiens (human)
hsa00620	Pyruvate metabolism - Homo sapiens (human)
hsa00630	Glyoxylate and dicarboxylate metabolism - Homo sapiens (human)
hsa00640	Propanoate metabolism - Homo sapiens (human)
hsa00650	Butanoate metabolism - Homo sapiens (human)
hsa00670	One carbon pool by folate - Homo sapiens (human)
hsa00730	Thiamine metabolism - Homo sapiens (human)
hsa00740	Riboflavin metabolism - Homo sapiens (human)
hsa00750	Vitamin B6 metabolism - Homo sapiens (human)
hsa00760	Nicotinate and nicotinamide metabolism - Homo sapiens (human)
hsa00770	Pantothenate and CoA biosynthesis - Homo sapiens (human)
hsa00780	Biotin metabolism - Homo sapiens (human)
hsa00785	Lipoic acid metabolism - Homo sapiens (human)
hsa00790	Folate biosynthesis - Homo sapiens (human)
hsa00830	Retinol metabolism - Homo sapiens (human)
hsa00860	Porphyrin and chlorophyll metabolism - Homo sapiens (human)
hsa00900	Terpenoid backbone biosynthesis - Homo sapiens (human)

hsa00910	Nitrogen metabolism - Homo sapiens (human)
hsa00920	Sulfur metabolism - Homo sapiens (human)
hsa00980	Metabolism of xenobiotics by cytochrome P450 - Homo sapiens (human)
hsa00982	Drug metabolism - cytochrome P450 - Homo sapiens (human)
hsa00983	Drug metabolism - other enzymes - Homo sapiens (human)
hsa01040	Biosynthesis of unsaturated fatty acids - Homo sapiens (human)

Table S2. Statistical comparison of pathway scores among the three groups. Data were expressed as mean ± standard deviation (SD)

pathway	C1	C2	C3	***P***
hsa00603	0.06±0.26	0±0.29	-0.06±0.29	0.003
hsa00604	0±0.28	-0.01±0.31	-0.02±0.3	0.88
hsa00512	0.04±0.24	-0.08±0.24	-0.03±0.27	9e−04
hsa00514	0.11±0.21	-0.04±0.22	-0.06±0.22	1.3e−10
hsa00230	0±0.14	-0.03±0.14	-0.04±0.15	0.057
hsa00564	0±0.14	-0.03±0.13	-0.04±0.16	0.12
hsa00565	0.03±0.16	-0.09±0.17	-0.08±0.19	2.8e−08
hsa00533	0.11±0.27	-0.06±0.25	-0.11±0.25	3.7e−11
hsa00601	0.1±0.21	-0.07±0.21	-0.08±0.26	8.5e−12
hsa00532	0.15±0.3	-0.08±0.31	-0.13±0.3	9.7e−14
hsa00534	0.12±0.22	0.01±0.24	-0.07±0.24	4.6e−09
hsa00511	0±0.28	0.11±0.29	-0.02±0.33	0.0016
hsa00531	-0.05±0.28	0.05±0.28	0.02±0.3	0.011
hsa00515	0.09±0.27	0.03±0.25	-0.12±0.24	5.2e−10
hsa00562	0.11±0.25	0±0.26	-0.12±0.28	1.5e−10
hsa00563	0.06±0.27	0.11±0.27	-0.08±0.33	5.8e−07
hsa00240	-0.01±0.16	0.02±0.19	-0.05±0.19	0.014
hsa00600	0.03±0.21	0±0.21	-0.09±0.21	5.3e−06
hsa00510	0.06±0.24	0.1±0.24	-0.08±0.26	1.8e−08
hsa00513	0.04±0.23	0.08±0.23	-0.05±0.25	6.3e−05
hsa00072	-0.44±0.26	-0.09±0.3	0.24±0.3	2.2e−16
hsa00280	-0.55±0.15	-0.05±0.29	0.33±0.24	2.2e−16
hsa00640	-0.5±0.16	-0.05±0.29	0.25±0.27	2.2e−16
hsa00380	-0.48±0.14	-0.09±0.21	0.3±0.19	2.2e−16
hsa00620	-0.45±0.14	0±0.2	0.27±0.17	2.2e−16
hsa00071	-0.54±0.17	-0.07±0.25	0.35±0.21	2.2e−16
hsa00410	-0.45±0.17	-0.06±0.26	0.29±0.2	2.2e−16
hsa00650	-0.49±0.18	-0.05±0.26	0.3±0.2	2.2e−16
hsa00120	-0.57±0.21	-0.03±0.35	0.35±0.27	2.2e−16
hsa00830	-0.43±0.16	-0.1±0.21	0.27±0.18	2.2e−16
hsa00140	-0.37±0.19	-0.02±0.22	0.25±0.18	2.2e−16
hsa00980	-0.42±0.19	-0.04±0.25	0.28±0.18	2.2e−16
hsa00982	-0.44±0.17	-0.06±0.24	0.29±0.19	2.2e−16
hsa00860	-0.3±0.22	-0.02±0.25	0.2±0.2	2.2e−16
hsa00040	-0.38±0.23	-0.01±0.27	0.23±0.22	2.2e−16
hsa00053	-0.42±0.23	-0.01±0.28	0.31±0.21	2.2e−16
hsa00430	-0.18±0.21	-0.11±0.27	0.09±0.24	2.2e−16
hsa00590	-0.13±0.19	-0.1±0.2	0.05±0.23	3e−11
hsa00591	-0.22±0.2	-0.13±0.22	0.1±0.22	2.2e−16
hsa00592	-0.13±0.2	-0.09±0.19	0.05±0.22	1.5e−12
hsa00910	-0.25±0.23	-0.06±0.27	0.18±0.26	2.2e−16

hsa00061	-0.31±0.2	0.03±0.23	0.18±0.24	2.2e−16
hsa00250	-0.3±0.15	-0.05±0.21	0.12±0.2	2.2e−16
hsa00330	-0.28±0.15	-0.07±0.19	0.18±0.17	2.2e−16
hsa00760	-0.24±0.17	-0.04±0.17	0.09±0.16	2.2e−16
hsa00790	-0.33±0.2	0.01±0.21	0.19±0.2	2.2e−16
hsa00020	-0.37±0.25	0.06±0.29	0.22±0.31	2.2e−16
hsa00130	-0.38±0.23	0±0.28	0.19±0.28	2.2e−16
hsa00340	-0.44±0.21	-0.09±0.26	0.26±0.22	2.2e−16
hsa00350	-0.43±0.14	-0.08±0.22	0.32±0.17	2.2e−16
hsa00360	-0.4±0.17	-0.06±0.29	0.28±0.22	2.2e−16
hsa00220	-0.45±0.23	-0.02±0.32	0.3±0.22	2.2e−16
hsa00260	-0.5±0.19	-0.04±0.28	0.29±0.2	2.2e−16
hsa00630	-0.48±0.18	-0.01±0.23	0.29±0.21	2.2e−16
hsa00190	-0.05±0.37	0.07±0.38	0.07±0.43	0.018
hsa00100	-0.15±0.39	0.12±0.37	0.07±0.4	6.8e−08
hsa00900	-0.14±0.29	0.1±0.3	0.02±0.3	3.2e−09
hsa00310	-0.14±0.2	0.05±0.24	0.03±0.26	1.5e−11
hsa00450	-0.2±0.21	0.06±0.25	0.09±0.26	2.2e−16
hsa00062	-0.21±0.21	0.04±0.24	0.09±0.24	2.2e−16
hsa01040	-0.29±0.23	0.04±0.26	0.13±0.25	2.2e−16
hsa00770	-0.26±0.23	-0.05±0.2	0.11±0.21	2.2e−16
hsa00270	-0.29±0.17	0.01±0.19	0.11±0.18	2.2e−16
hsa00670	-0.24±0.18	-0.03±0.2	0.08±0.21	2.2e−16
hsa00520	-0.02±0.21	0.03±0.22	-0.05±0.24	0.038
hsa00051	-0.15±0.21	-0.02±0.2	0.02±0.23	7.9e−10
hsa00052	-0.05±0.22	-0.02±0.21	0.04±0.22	0.0043
hsa00500	-0.16±0.19	-0.05±0.22	0.05±0.21	2.6e−13
hsa00010	-0.27±0.14	-0.03±0.16	0.14±0.18	2.2e−16
hsa00561	-0.19±0.14	0±0.17	0.08±0.17	2.2e−16
hsa00730	-0.13±0.23	0±0.26	0.08±0.23	1.9e−11
hsa00030	-0.22±0.2	0.03±0.2	0.08±0.22	2.2e−16
hsa00480	-0.16±0.21	0.01±0.23	0.05±0.21	1.4e−14
hsa00983	-0.29±0.17	-0.01±0.2	0.14±0.16	2.2e−16

Table S3: Background and clinical characteristics of subtype HCC patients from TCGA

		C1(n=129)	C2(n=117)	C3(n=128)	*P*
**Survival status**	alive	83(64.3%)	70(59.8%)	95(74.2%)	0.049
	dead	46(35.7%)	47(40.2%)	33(25.8%)	
**Age**	≤60	75(58.1%)	53(45.3%)	49(38.3%)	0.012
	>60	53(41.1%)	64(54.7%)	79(61.7%)	
	unknow	1(0.8%)	0(0%)	0(0%)	
**Gender**	male	70(54.3%)	84(71.8%)	99(77.3%)	<0.001
	female	59(45.7%)	33(28.2%)	29(22.7%)	
**Grade**	G1	11(8.5%)	14(12%)	29(22.7%)	<0.001
	G2	48(37.2%)	55(47%)	75(58.6%)	
	G3	62(48.1%)	42(35.9%)	21(16.4%)	
	G4	6(4.7%)	4(3.4%)	2(1.6%)	
	unknow	2(1.6%)	2(1.7%)	1(0.8%)	
**Stage**	Stage I	11(8.5%)	51(43.6%)	75(58.6%)	0.016
	Stage II	48(37.2%)	25(21.4%)	25(19.5%)	
	Stage III	62(48.1%)	31(26.5%)	17(13.3%)	
	Stage IV	6(4.7%)	1(0.9%)	2(1.6%)	
	unknow	2(1.6%)	9(7.7%)	9(7%)	
**T**	T1	48(37.2%)	54(46.2%)	81(63.3%)	0.005
	T2	41(31.8%)	28(23.9%)	25(19.5%)	
	T3	36(27.9%)	26(22.2%)	19(14.8%)	
	T4	4(3.1%)	7(6%)	2(1.6%)	
	TX	0(0%)	1(0.9%)	1(0.8%)	
	unknow	0(0%)	1(0.9%)	(0%)	
**N**	N0	95(73.6%)	77(65.8%)	82(64.1%)	0.231
	N1	2(1.6%)	2(1.7%)	46(35.9%)	
	NX	32(24.8%)	38(32.5%)	0(0%)	
**M**	M0	100(77.5%)	88(75.2%)	80(62.5%)	0.043
	M1	2(1.6%)	29(24.8%)	2(1.6%)	
	MX	27(20.9%)	0(0%)	46(35.9%)	

(n = 374)

Table S4: Association of risk score with Somatic Variants.

gene	C3-wild	C3-mutation	C1-wild	C1-mutation	pvalue
CTNNB1	77(63.11%)	45(36.89%)	116(92.8%)	9(7.2%)	4.03E-08
TP53	112(91.8%)	10(8.2%)	89(71.2%)	36(28.8%)	6.47E-05
APOB	112(91.8%)	10(8.2%)	120(96%)	5(4%)	0.265159
TTN	95(77.87%)	27(22.13%)	104(83.2%)	21(16.8%)	0.369269
LRP1B	117(95.9%)	5(4.1%)	116(92.8%)	9(7.2%)	0.436105
PCLO	106(86.89%)	16(13.11%)	113(90.4%)	12(9.6%)	0.5026
HMCN1	113(92.62%)	9(7.38%)	119(95.2%)	6(4.8%)	0.560964
RYR2	113(92.62%)	9(7.38%)	113(90.4%)	12(9.6%)	0.690553
XIRP2	115(94.26%)	7(5.74%)	120(96%)	5(4%)	0.734524
ABCA13	113(92.62%)	9(7.38%)	118(94.4%)	7(5.6%)	0.757494
CUBN	117(95.9%)	5(4.1%)	118(94.4%)	7(5.6%)	0.800394
DOCK2	116(95.08%)	6(4.92%)	117(93.6%)	8(6.4%)	0.819336
FAT3	116(95.08%)	6(4.92%)	117(93.6%)	8(6.4%)	0.819336
CSMD3	116(95.08%)	6(4.92%)	117(93.6%)	8(6.4%)	0.819336
MUC16	105(86.07%)	17(13.93%)	107(85.6%)	18(14.4%)	1
SDK1	116(95.08%)	6(4.92%)	119(95.2%)	6(4.8%)	1
CACNA1E	114(93.44%)	8(6.56%)	116(92.8%)	9(7.2%)	1
HERC2	116(95.08%)	6(4.92%)	119(95.2%)	6(4.8%)	1
ADGRV1	116(95.08%)	6(4.92%)	119(95.2%)	6(4.8%)	1
OBSCN	115(94.26%)	7(5.74%)	118(94.4%)	7(5.6%)	1
FLG	113(92.62%)	9(7.38%)	116(92.8%)	9(7.2%)	1

Table S5. Univariate Cox regression analysis for key enzyme of FAO pathway

id	HR	HR.95L	HR.95H	pvalue
ACADL	0.803034	0.676844	0.952751	0.011907
ACADM	1.00882	0.849697	1.197741	0.920137
CPT1A	0.938902	0.783904	1.124547	0.493438
CPT1B	0.98885	0.585008	1.671471	0.966605
CPT1C	1.248715	0.787	1.981308	0.345672

Table S6 Potential TF and binding sites of TEAD4 on the promoter of ACADL

1. Potential TF predicted by hTF database

No. of datasets	TF	Tissue	No. of peaks (total/average)	No. of peaks in gene body (total/average)	No. of peaks around TSS (total/average)
1	CEBPA	Liver	6/ 6	5 / 5	1 / 1
2	CEBPB	Liver	6/ 3	4 / 2	2 / 1
1	EED	Liver	3/ 3	2 / 2	1 / 1
1	ELF1	Liver	2/ 2	1 / 1	1 / 1
1	FOXA1	Liver	2/ 2	1 / 1	1 / 1
1	FOXA2	Liver	2/ 2	1 / 1	1 / 1
3	HNF4A	Liver	10/ 3	5 / 1	5 / 1
1	HNF4G	Liver	3/ 3	2 / 2	1 / 1
1	MAFF	Liver	2/ 2	2 / 2	0 / 0
1	TEAD4	Liver	2/ 2	2 / 2	0 / 0
1	YY1	Liver	2/ 2	1 / 1	1 / 1

2. Potential transcription factor binding sites of TEAD4 on the promoter of ACADL

Matrix ID	Name	Score	Relative score	Start	End	Strand	Region	Predicted sequence
MA0809.2	TEAD4	15.74874	0.990949	66	77	+	R1	acacattccaga
MA0809.2	TEAD4	9.511594	0.870617	468	479	-	R2	tgacatttcaac
MA0809.1	TEAD4	9.721458	0.927736	935	944	-	R3	agcattcttt

Table S7. Baseline characteristics of 78 patients with HCC.

Vairbale	Categories	Number of patients
Age(years)	<50	46(58.97%)
	≥50	32(41.03%)
Gender	Female	5(6.41%)
	male	73(93.59%)
Differentiation	high	11(14.1%)
	low	67(85.9%)
Tumor size(cm)	<5	28(35.9%)
	≥5	50(64.1%)
Tumor number	Single	73(93.6%)
	Multiple	5(6.4%)
Vascular Invasion	No	20(25.64%)
	Yes	58(74.36%)
Cirrhosis	No	41(52.56%)
	Yes	37(47.44%)
AFP (ng/ml)	<400	34(43.59%)
	≥400	44(56.41%)
ALT(U/L)	<40	26(33.33%)
	≥40	52(66.67%)
HBsAg	No	65(83.33%)
	Yes	13(16.67%)
Collagen	low	40(51.28%)
	high	38(48.72%)
ACADL	low	38(48.72%)
	high	40(51.28%)

Table S8. Univariate and multivariate analysis of factors associated with the OS in HCC patients

	Categories	Univariate analysis		Multivariate analysis	
		**HR (95% CI)**	***P***	**HR (95% CI)**	***P***
age	≥50/ <50	1.081(0.681-1.715)	0.742		
gender	Male/Female	0.516(0.203-1.309)	0.163		
Differentiation	Low/High	1.964(0.956-4.032)	0.066	1.61(0.685-3.782)	0.275
Tumor size(cm)	≥5/ <5	1.105(0.686-1.781)	0.682		
Tumor number	Single/Multiple	0.419(0.152-1.154)	0.093	0.672(0.22-2.052)	0.485
Vascular invasion	Yes/No	1.869(1.056-3.307)	0.032	0.996(0.478-2.077)	0.991
Cirrhosis	Yes/No	0.877(0.556-1.383)	0.572		
AFP	≥400/ <400	1.378(0.87-2.182)	0.172		
ALT	≥40/ <40	1.141(0.701-1.859)	0.596		
HBsAg	Yes/No	2.7(1.438-5.068)	0.002	2.284(1.12-4.657)	0.023
collagen	High/low	5.553(3.257-9.466)	<0.001	3.829(2.193-6.685)	<0.001
ACADL	High/low	0.349(0.215-0.567)	<0.001	0.406(0.241-0.686)	0.001
$collagen^{high}$/$ACADL^{low}$	$collagen^{high}ACADL^{low}$ / $collagen^{low}ACADL^{high}$	6.637(3.347-13.67)	<0.001	4.912(2.392-10.08)	

P < 0.05 was regarded as statistically significant and P value was calculated by Cox proportional hazards regression.

Table S9. Sequences of Real time-PCR primers and siRNA

Real time-PCR primers:

Gene	Forward Primer	Reverse Primer
ACADL	TTGGCAAAACAGTTGCTCAC	ACATGTATCCCCAACCTCCA
CYR61	ATGGTCCCAGTGCTCAAAGA	GGGCCGGTATTTCTTCACAC
ACADL-Region1	GAGCCTAGGAGGGCTAAAAGCA	AAGGCTGAGGGTTGGGAAGAA
ACADL-Region2	CACATTTGGTTAGCATGTTTATCAGTTTT	GGCTTAAACTATCTGGGTAGTTTGCTTG
ACADL-Region3	CCATAGCTTTCATCCGTTACTCAAAGC	CAGCAATAGGATTTTACAGCGAAACTG

The siRNA sequences are as follows:

YAP1 siRNA: GGUGAUACUAUCAACCAAA
